# Breaking the ROS inflammation loop: a hierarchical nanozyme natural enzyme cascade for acetaminophen induced acute liver injury

**DOI:** 10.1186/s12951-026-04912-2

**Published:** 2026-08-10

**Authors:** Mengmeng Dong, Xiaomin Huang, Kaiqiang Li, Xiaorong Zhu, Tingting Hu, Xiaohuan Guo, Ke Hao, Fei Kong, Mei Yang, Ning Hu, Yingwei Wang, Lisen Lu, Zhen Wang

**Affiliations:** 1 Laboratory Medicine Center, Department of Transfusion Medicine, Affiliated People’s Hospital, Zhejiang Provincial People’s Hospital, Hangzhou Medical College, Hangzhou, 310014 China; 2 NHC Key Laboratory of Tropical Disease Control, School of Life Sciences and Medical Technology, Hainan Medical University, Haikou, Hainan, 571199 China; 3 Eye Institute, Department of Ophthalmology, Eye & ENT Hospital, Key Laboratory of Myopia, Fudan University, Chinese Academy of Medical Sciences, Shanghai, 200000 China; 4 School of Laboratory Medicine, Hangzhou Medical College, Hangzhou, 310053 China; 5 General Surgery Department, Children’s Hospital, School of Medicine, Zhejiang University, National Clinical Research Center for Children’s Health, Hangzhou, 310052 China; 6 Center for Laboratory Medicine, Precision Medicine Center, Tiantai People’s Hospital of Zhejiang Province (Tiantai Branch of Zhejiang Provincial People’s Hospital), Hangzhou Medical College, Taizhou, 317200 China

**Keywords:** SOD, Metal-organic frameworks, Catalytic hierarchy, ROS cascade interruption, Acute liver injury

## Abstract

Acetaminophen (APAP) induced acute liver injury (ALI) is driven by a self amplifying cascade of reactive oxygen species (ROS) and lipid peroxidation, yet current therapies fail to interrupt this pathological loop. Here we engineer a liver targeted hierarchical nanozyme, SOD@ZIF-8/Mn_3_O_4_, which integrates natural superoxide dismutase (SOD) and Mn_3_O_4_ nanozymes within a ZIF‑8 nanoplatform. Unlike conventional single step scavengers, this system implements a catalytic hierarchy matching strategy. SOD rapidly converts O_2_·^−^ to H_2_O_2_, while Mn_3_O_4_ simultaneously decomposes H_2_O_2_ and intercepts ·OH, thereby converting the entire ROS cascade into benign water. Density functional theory calculations reveal that the SOD–Mn_3_O_4_ interface lowers the energy barriers for catalase like and glutathione peroxidase like steps, establishing a synergistic channeling effect. In APAP injured AML-12 cells and a murine ALI model, the nanozyme not only alleviates oxidative stress and lipid peroxidation but also breaks the ROS inflammation feed forward loop by restoring the Keap1/Nrf2/HO‑1 redox switch and suppressing NF‑κB activation. Transcriptomic and functional analyses confirm that treatment rewires hepatic transcriptional programs associated with lipid metabolism and inflammatory signaling. This work establishes a modular nanozyme natural enzyme hybrid platform where the MOF acts as a spatiotemporal organizer, transforming the pathological ROS chain into a controlled detoxification pathway. The concept is readily extensible to other diseases driven by sequential ROS cascades, offering a paradigm shift from single enzyme mimicry to pathway reprogramming nanobiocatalysis.

**Graphical abstract:**

**Figure:**
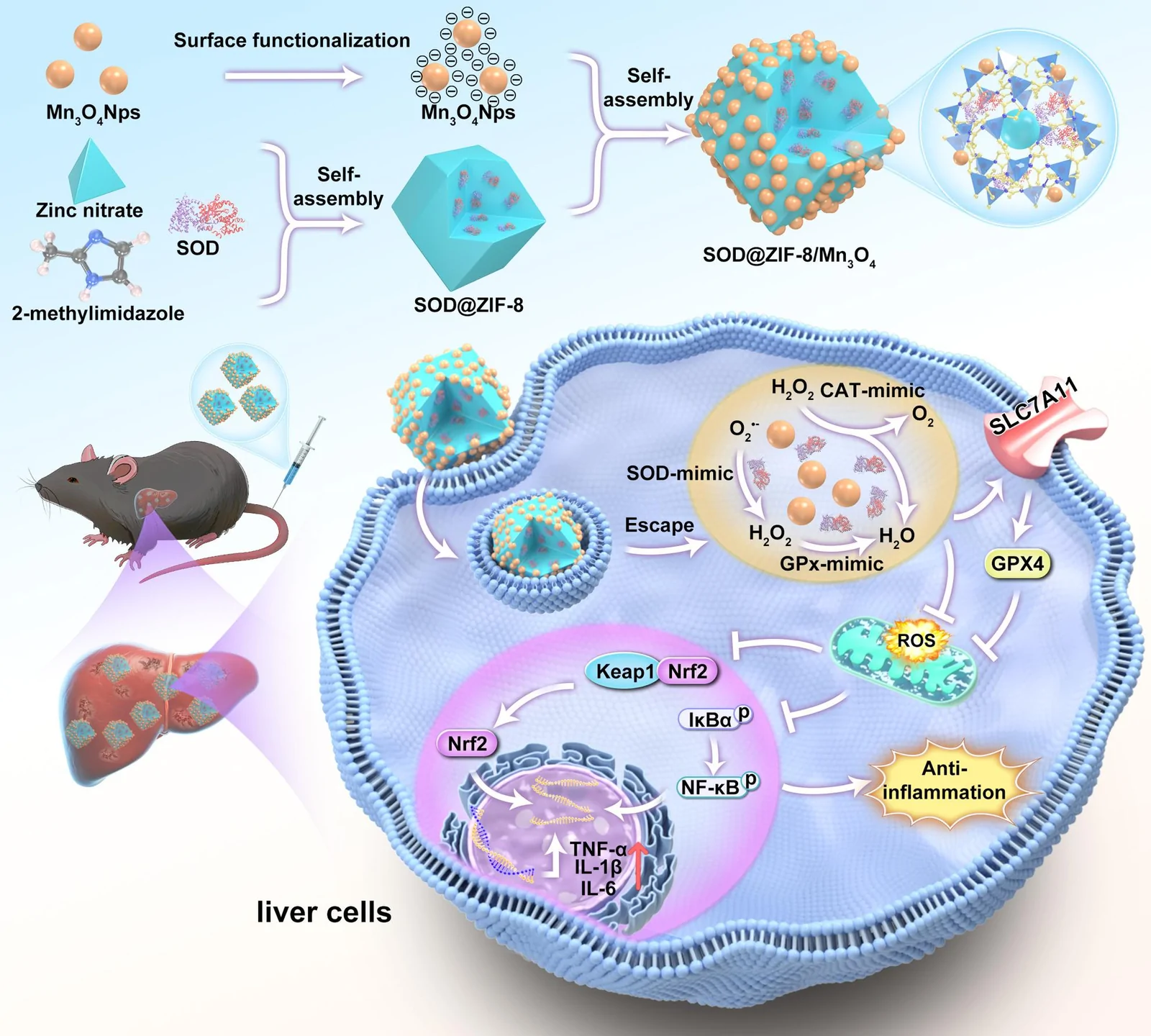

**Supplementary Information:**

The online version contains supplementary material available at https://doi.org/10.1186/s12951-026-04912-2.

## Introduction

Acute liver injury (ALI) is a severe and rapidly progressing liver condition characterized by extensive hepatocellular necrosis and impaired liver function [1]. Clinically, ALI is frequently induced by drug overdose, with acetaminophen (APAP) implicated in approximately 60 to 70% of cases. Globally, APAP poisoning accounts for over 100,000 incidents annually, with in hospital mortality rates reaching 30%, underscoring its high lethality and substantial socioeconomic impact [2]. The global incidence of ALI is increasing annually, with the continued prevalence of APAP excess as a significant cause of liver injury emphasizing the significance of this clinical problem. Currently, therapeutic options for ALI remain limited, primarily consisting of antioxidant drug therapy or liver transplantation [3]. N‑Acetylcysteine (NAC) is the primary clinical antidote for ALI. However, its utility is constrained by a narrow therapeutic time window, limited efficacy in modulating the lipid peroxidation pathway in advanced injury, and the inherent challenges of liver transplantation, including donor shortages and high costs [4–6]. Consequently, the development of novel therapeutic strategies that are both efficient and capable of precisely modulating oxidative stress and inflammatory responses is crucial for the development of effective clinical interventions for ALI.

Intracellular superoxide dismutase (SOD) is a pivotal endogenous antioxidant enzyme that catalyzes the dismutation of the superoxide anion (O_2_·^−^) into hydrogen peroxide (H_2_O_2_) and oxygen. This reaction is the primary cellular defense against superoxide, mitigating the formation of more damaging reactive species like peroxynitrite (ONOO⁻) and subsequent oxidative cellular damage [7, 8]. The pathological microenvironment in ALI is defined by a rapid and excessive accumulation of O_2_·^−^ and H_2_O_2_, where oxidative stress acts as a principal driver of disease progression. Therefore, therapeutic strategies aimed at augmenting SOD activity to catalytically neutralize these ROS and facilitate their conversion into water represent a highly promising approach for mitigating oxidative damage and improving outcomes in ALI [9–11]. Despite its high catalytic specificity and potent antioxidant capacity, the clinical translation of native SOD is hampered by several intrinsic limitations. These include rapid clearance from sites of inflammation, poor in vivo stability, and a lack of targeted delivery capability [12].

Recent investigations have demonstrated the therapeutic potential of exogenous SOD in mitigating ROS and ameliorating pathology in various inflammatory diseases, including osteoarthritis (OA) [13], rheumatoid arthritis [14], and gastroenteritis [15]. However, its specific application and efficacy in the context of ALI remain largely unexplored. The development of advanced nano delivery systems designed to enhance the stability, targeted accumulation, and bioavailability of SOD at hepatic injury sites is therefore crucial for unlocking its full therapeutic potential for ALI. In parallel, nanomaterials exhibiting intrinsic enzyme mimetic activities, known as nanozymes, have emerged as promising alternatives or supplements to natural enzymes. These nanozymes offer distinct advantages, including superior stability, lower production costs, and tunable multifunctionality [16, 17]. Among various nanozymes, those based on metal oxides, particularly manganese based nanoparticles such as Mn_3_O_4_, have attracted significant research interest [18]. Mn_3_O_4_ nanoparticles exhibit a remarkable repertoire of enzyme mimetic activities, including those resembling superoxide dismutase (SOD), catalase (CAT), and glutathione peroxidase (GPX) [19]. This multi enzyme profile enables Mn_3_O_4_ to efficiently scavenge a broad spectrum of ROS and has also been shown to effectively inhibit the process of lipid peroxidation, a form of iron dependent cell death [20]. Consequently, Mn_3_O_4_ nanozymes have demonstrated significant therapeutic efficacy in various models of oxidative stress related diseases, such as osteoarthritis [21] and ischemic stroke [22]. A particularly advantageous property for liver targeted therapy is the innate propensity of manganese based nanoparticles to accumulate in hepatic tissue. This is facilitated primarily by efficient uptake via hepatic macrophages (Kupffer cells) and the hepatic vascular system [23, 24]. Therefore, Mn_3_O_4_ is anticipated to achieve high local concentrations in the liver and exert sustained antioxidant effects [25], presenting a highly promising strategy for the prevention and treatment of ALI.

Metal organic frameworks (MOFs), a class of porous crystalline materials formed by the coordination of metal ions with organic linkers, have recently emerged as highly promising platforms for enzyme immobilization and delivery [26, 27]. Their tunable porosity, exceptionally high surface area, and structural versatility facilitate the efficient encapsulation of biomacromolecules like enzymes, while simultaneously protecting them from degradation and enhancing their stability [28]. These attributes make MOFs attractive candidates for constructing advanced biocatalytic systems for a range of biomedical applications [29, 30]. Zeolitic imidazolate framework‑8 (ZIF‑8), a subclass of MOFs self assembled from zinc ions and 2‑methylimidazole ligands, is particularly notable for its excellent biocompatibility and mild synthesis conditions [31]. ZIF‑8 nanoparticles can encapsulate a diverse range of biomacromolecules, including proteins, enzymes, and nucleic acids, via a straightforward and biomimetic mineralization process that effectively preserves bioactivity and minimizes premature leakage [32]. Furthermore, a key feature of ZIF‑8 is its degradation under mildly acidic conditions, which enables the targeted release of encapsulated cargo in the acidic microenvironments typically associated with inflamed tissues, such as injured liver [33]. Collectively, these properties establish ZIF‑8 as an ideal candidate for constructing sophisticated multi enzyme cascade catalytic platforms [34].

**Scheme 1:**
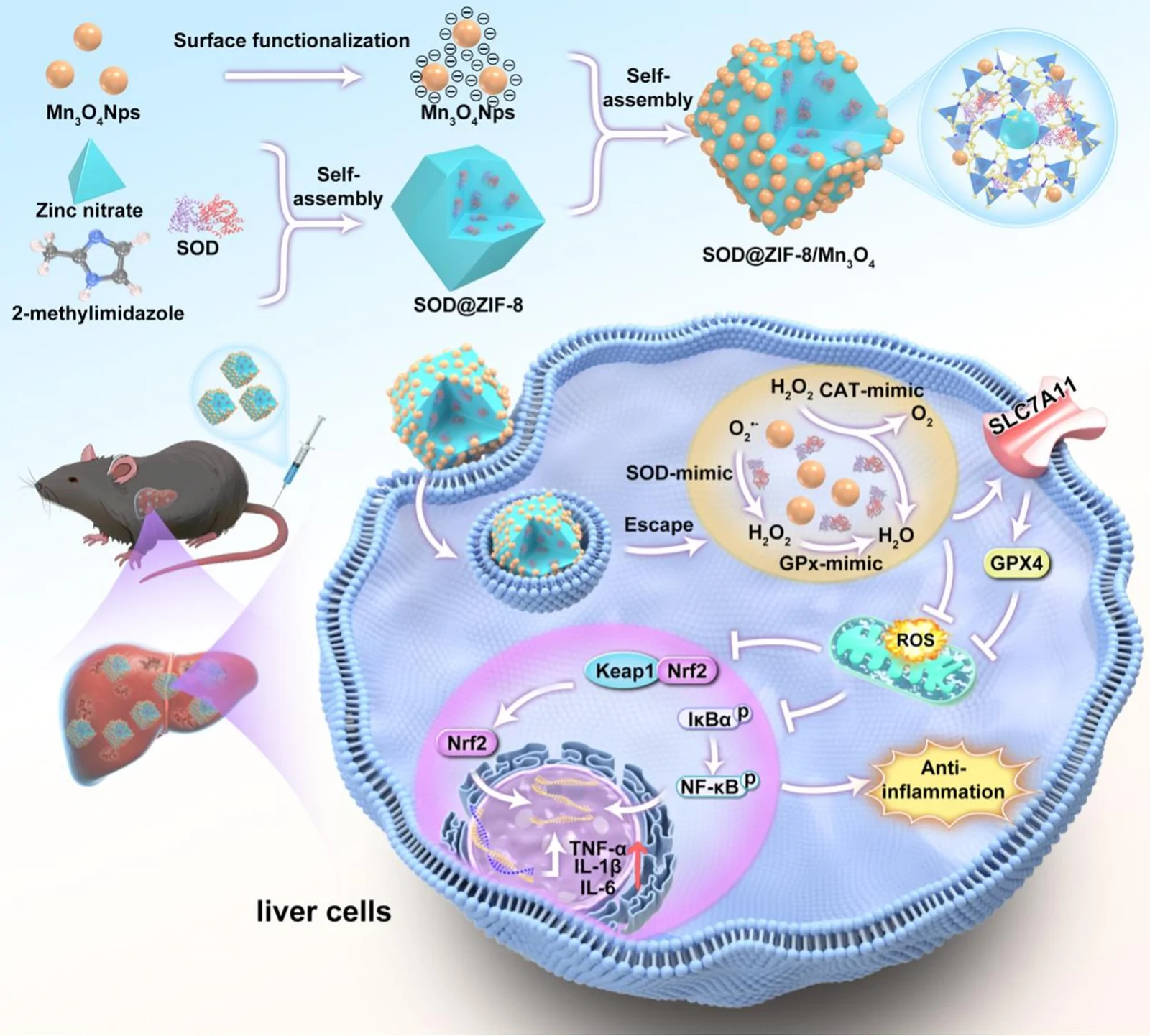
The nanocomposite was constructed by encapsulating superoxide dismutase (SOD) within a zeolitic imidazolate framework-8 (ZIF-8) and further functionalizing its surface with Mn_3_O_4_ nanoparticles. Upon intravenous administration, the nanozyme preferentially accumulates in the liver, where it exhibits multi-enzyme mimetic activities (SOD-, CAT-, and GPX-like) and scavenges ROS, thereby mitigating oxidative stress, lipid peroxidation, and inflammation, and ultimately protecting against hepatocyte damage

Despite this promise, the application of such ZIF‑8 based cascade systems for the treatment of hepatic injury remains largely unexplored. More importantly, APAP induced ALI features a ROS burst cascade that converts O_2_·^−^ to H_2_O_2_ and then to ·OH, demanding sequential rather than single step scavenging. Conventional single component antioxidants often fail because they either leave H_2_O_2_ unprocessed, allowing pro oxidant side reactions, or cannot intercept the initial O_2_·^−^ surge. Herein, we propose a catalytic hierarchy matching strategy. SOD rapidly converts O_2_·^−^ to H_2_O_2_, while Mn_3_O_4_ simultaneously decomposes H_2_O_2_ and intercepts ·OH, thereby avoiding the pro oxidant risk of H_2_O_2_ accumulation. This design transforms the pathological ROS chain into a benign output (water), representing a paradigm shift from single enzyme mimicry to pathway reprogramming nanobiocatalysis. To implement this concept, we rationally constructed a synergistic antioxidant cascade nanozyme by co integrating natural SOD and multifunctional Mn_3_O_4_ nanozyme within a ZIF‑8 nanocarrier. The composite system, termed SOD@ZIF‑8/Mn_3_O_4_, was engineered to comprehensively mitigate oxidative stress and associated inflammatory responses in APAP induced ALI. As illustrated in Scheme 1, our design leverages the ZIF‑8 framework to significantly enhance the stability of the encapsulated SOD, shield it from proteolytic degradation, and facilitate its responsive release within the acidic and oxidative microenvironment of the injured liver. Simultaneously, the incorporated Mn_3_O_4_ nanozymes endow the platform with robust multi enzyme mimetic activities (SOD like, CAT like, GPX like), enabling it to scavenge a wide spectrum of ROS through a synergistic catalytic cascade. We hypothesize that this cooperative antioxidant strategy will not only achieve superior total ROS scavenging efficiency but also enable the effective modulation of key downstream oxidative and inflammatory pathways critically involved in ALI progression, ultimately breaking the ROS inflammation feed forward loop.

## Results and discussion

### Synthesis and characterization of SOD@ZIF-8/Mn_3_O_4_

**Fig. 1:**
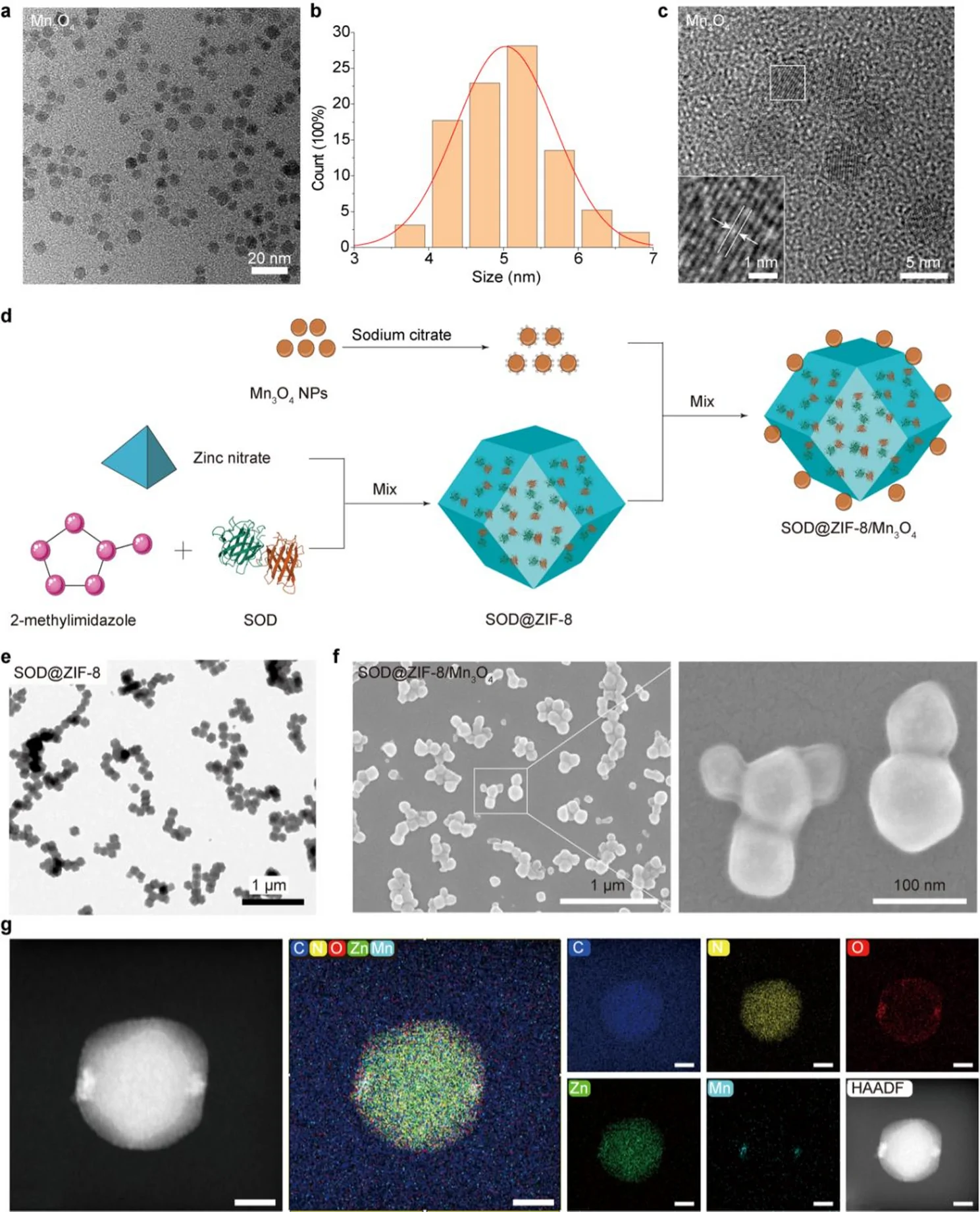
Synthesis and structural characterization of SOD@ZIF-8/Mn_3_O_4_ nanocomposites. (**a**) TEM image of synthesized Mn_3_O_4_ nanoparticles (NPs). (**b**) Size distribution histogram of Mn_3_O_4_ NPs. (**c**) HRTEM image showing lattice fringes corresponding to the (211) plane of tetragonal Mn_3_O_4_. (**d**) Schematic representation of the step-wise synthesis of SOD@ZIF-8/Mn_3_O_4_. (**e**) TEM image of SOD@ZIF-8 NPs showing retained rhombic dodecahedral morphology. (**f**) SEM image of the final SOD@ZIF-8/Mn_3_O_4_ composite. (**g**) STEM image and corresponding elemental mapping (Zn, N, O, Mn, and C) confirming homogeneous distribution of all components. Scale bar: 20 nm

Mn_3_O_4_ nanoparticles were prepared via a hydrothermal method and subsequently characterized using multiple techniques. High resolution transmission electron microscopy (HRTEM) was initially employed to observe the morphology and particle size of the resulting Mn_3_O_4_ NPs (Fig. 1a). The particles exhibited uniform dispersion and well defined morphology, with an average diameter of 5.41 nm (Fig. 1b). The HRTEM image exhibited clear lattice fringes with a spacing of 0.25 nm, which corresponds to the (211) plane of tetragonal Mn_3_O_4_ (JCPDS No. 24–0734), confirming the crystalline structure of the product (Fig. 1c). The SOD@ZIF-8/Mn_3_O_4_ nanocomposite was subsequently fabricated via a two step procedure (Fig. 1d). First, SOD was efficiently encapsulated within the ZIF-8 framework via a facile biomimetic mineralization process to form SOD@ZIF-8. Transmission electron microscopy (TEM) analysis confirmed that the resulting SOD@ZIF-8 particles maintained the characteristic rhombic dodecahedral morphology of ZIF-8 with uniform dispersion (Fig. 1e), indicating that the encapsulation process did not compromise the structural integrity of the MOF. In the second step, citrate functionalized Mn_3_O_4_ NPs were anchored onto the surface of the pre formed SOD@ZIF-8 to yield the final SOD@ZIF-8/Mn_3_O_4_ composite. Scanning electron microscopy (SEM) imaging revealed a transition from the smooth surfaces of pristine ZIF-8 to a rougher texture for the composite (Fig. 1f), suggesting successful surface modification with Mn_3_O_4_ NPs. HRTEM confirmed the final composite size to be approximately 50 nm (Figure S1, Supporting information). Energy dispersive X-ray spectroscopy (EDS) elemental mapping unequivocally confirmed the homogeneous distribution of Zn (from ZIF-8), Mn (from Mn_3_O_4_), O, N, and C throughout the nanocomposite structure (Fig. 1g), providing direct evidence for the successful integration of all components. Inductively coupled plasma optical emission spectrometry (ICP-OES) quantified the Mn content at 0.449 mg/mL. The encapsulation efficiency (EE%) and loading content (LC%) of SOD were determined to be 58% and 8.29%, respectively (Figure S2, Supporting information). Collectively, these data confirm the successful fabrication of the SOD@ZIF-8/Mn_3_O_4_ nanocomposite.

**Fig. 2:**
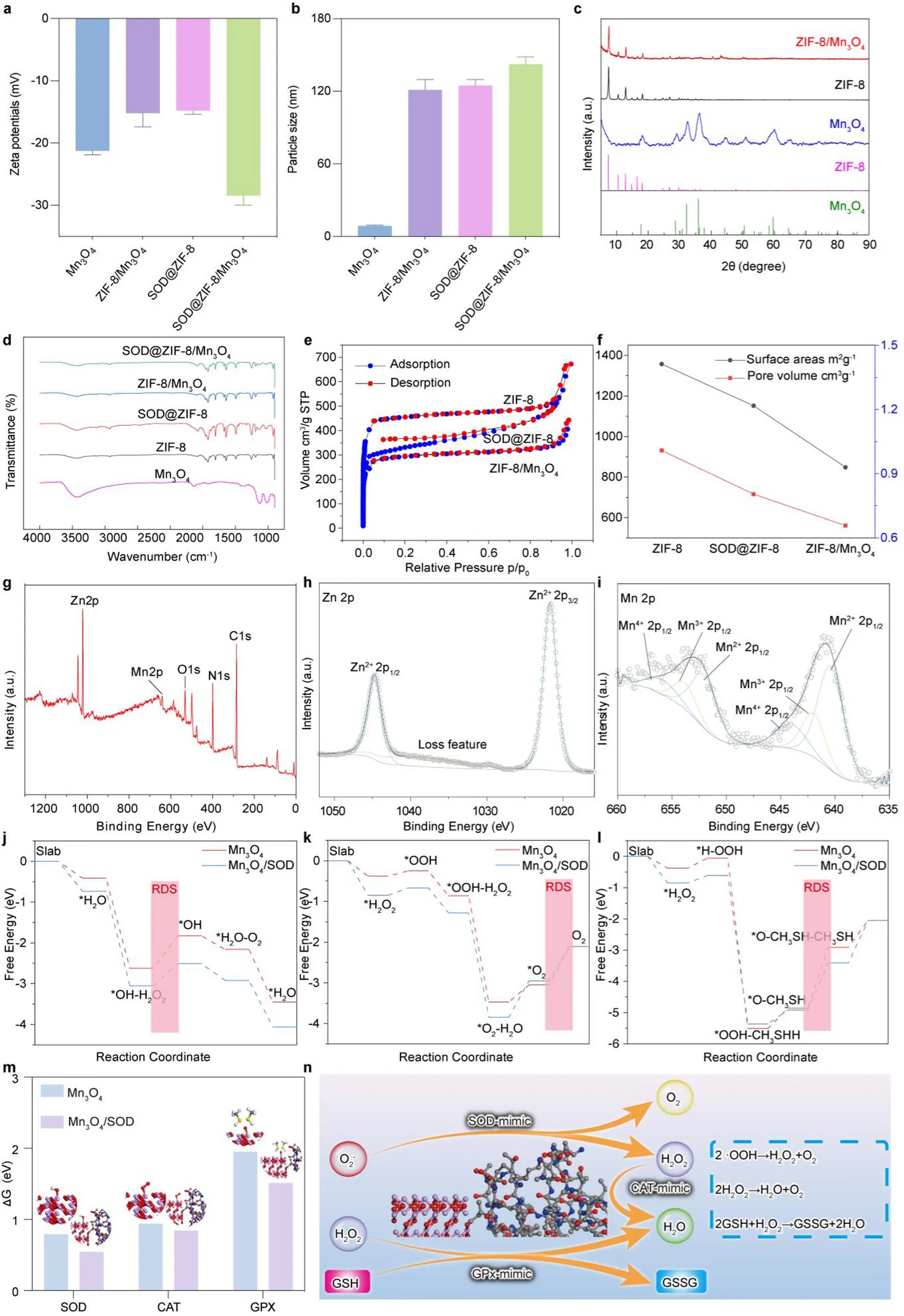
Physicochemical and catalytic characterization of SOD@ZIF-8/Mn_3_O_4_. (**a**) Zeta potential of the nanocomposite in aqueous dispersion and (**b**) hydrodynamic size distribution (*n* = 3). (**c**) XRD patterns of ZIF-8, Mn_3_O_4_, and SOD@ZIF-8/Mn_3_O_4_. (**d**) FTIR spectra confirming successful integration of SOD and Mn_3_O_4_. (**e**) N_2_ adsorption-desorption isotherms and (**f**) corresponding pore size distributions. (**g**) XPS survey spectrum and high-resolution spectra of (**h**) Zn 2p and (**i**) Mn 2p. (**j–l**) Gibbs free energy diagrams for (**j**) SOD-like, (**k**) CAT-like, and (**l**) GPX-like pathways from DFT calculations. (**m**) Energy barriers of the rate-determining steps (RDS) for each pathway. (**n**) Schematic of the proposed cascade catalytic mechanism

Following the morphological analysis, the hydrated particle size and zeta potential of the synthesized nanoparticles were measured. Zeta potential measurements (Fig. 2a) showed that Mn_3_O_4_, ZIF-8/Mn_3_O_4_, SOD@ZIF-8, and SOD@ZIF-8/Mn_3_O_4_ all exhibited negative surface charges. Notably, the zeta potential of SOD@ZIF-8/Mn_3_O_4_ was lower than that of SOD@ZIF-8, further supporting successful Mn_3_O_4_ surface modification and confirming the formation of the hybrid composite. As shown in Fig. 2b, ZIF-8 nanoparticles exhibited an average hydrodynamic diameter of about 120 nm as measured by DLS. The particle size of ZIF-8 slightly increased after SOD encapsulation, likely due to the aggregation of enzyme molecules into clusters, which were subsequently coated by the ZIF-8 framework. The hydrodynamic diameter of the Mn_3_O_4_ NPs was measured to be about 10 nm. Following the surface attachment of Mn_3_O_4_, the final SOD@ZIF-8/Mn_3_O_4_ composite exhibited a further increase in hydrodynamic diameter to around 130 nm, consistent with successful surface functionalization. The larger DLS-measured size compared with the TEM-observed particle size is mainly due to the hydrated layer, surface-adsorbed molecules, and slight solution-state aggregation of the nanocomposite. X-ray diffraction (XRD) patterns confirmed the co existence of crystalline phases from both ZIF-8 and Mn_3_O_4_ in the composite material, indicating that the structural integrity of the ZIF-8 framework was maintained after Mn_3_O_4_ integration (Fig. 2c). Fourier transform infrared (FTIR) spectroscopy provided further evidence for successful composite formation (Fig. 2d). The enhanced intensity of peaks at 1583 cm⁻¹ (C = N stretch) and 1308 cm⁻¹ (C-N vibration) in SOD@ZIF-8/Mn_3_O_4_, compared to ZIF-8, confirmed the presence of encapsulated SOD. Furthermore, the appearance of a distinct peak at 528 cm⁻¹, attributable to Mn-O vibrations, served as direct evidence for the successful incorporation of Mn_3_O_4_ into the nanocomposite. The emergence of a peak at 1421 cm⁻¹, associated with carboxylate groups from the citrate coating on Mn_3_O_4_, suggests coordination between the nanoparticle surface and the ZIF-8 framework. Collectively, the FTIR spectra corroborate the successful synthesis of the ternary SOD@ZIF-8/Mn_3_O_4_ nanocomposite.

N_2_ adsorption desorption isotherms were measured to analyze the textural properties of the nanomaterials (Figs. 2e-f; Table S2, Supporting information). The specific surface area of pristine ZIF-8 was 1386.41 m^2^/g, which decreased significantly to 877.53 m^2^/g for the ZIF-8/Mn_3_O_4_ composite. A concomitant reduction in pore volume from 1.03649 cm^3^/g to 0.68477 cm^3^/g was also observed. This substantial decrease in both surface area and pore volume provides strong evidence for the successful surface modification and partial pore blockage by the incorporated Mn_3_O_4_ nanoparticles and SOD enzyme. The enzyme mimetic activity of Mn_3_O_4_ originates from the mixed valence states of manganese (Mn^2+^ and Mn^3+^), which facilitate redox cycling. X-ray photoelectron spectroscopy (XPS) was employed to analyze the chemical states of elements in the composite. The survey spectrum confirmed the presence of Zn, Mn, O, N, and C (Fig. 2g). High resolution spectra of Zn 2p showed peaks at 1021.8 eV (2p_3/2_) and 1044.9 eV (2p_1/2_), characteristic of Zn^2+^ in ZIF-8 (Fig. 2h). The Mn 2p spectrum (Fig. 2i) exhibited two major peaks for Mn 2p_3/2_ at binding energies of 641.97 eV and 644.07 eV, corresponding to Mn^2+^ and Mn^3+^ species, respectively. The coexistence of these two oxidation states confirms the redox active nature of the Mn_3_O_4_ component, which is crucial for its multi enzyme mimetic activities.

To evaluate the long term stability of SOD@ZIF-8/Mn_3_O_4_ under physiologically relevant conditions, the nanocomposite was dispersed in DMEM medium containing 10% FBS. Its hydrodynamic diameter, PDI and zeta potential were monitored by DLS on days 0, 3, 5, and 7. As shown in Table S3, the hydrodynamic diameter of the nanozyme remained within the range of approximately 130 to 150 nm throughout the 7 day storage period, with no significant increase observed. The PDI values remained consistently below 0.3, indicating a uniform size distribution and the absence of significant aggregation. Additionally, the zeta potential remained stable between −28 mV and −30 mV, suggesting that the surface charge was well maintained over time. The hydrodynamic size of SOD@ZIF-8/Mn_3_O_4_ showed no obvious change in H_2_O, PBS, or DMEM over 7 days, indicating its favorable colloidal stability in different media (Figure S3, supporting information). These results demonstrate that SOD@ZIF-8/Mn_3_O_4_ possesses excellent colloidal stability in serum containing medium, providing a crucial foundation for its subsequent in vitro and in vivo applications.

To gain insight into the reaction mechanisms and synergistic effects, we performed Density Functional Theory (DFT) calculations to model the enzyme mimetic catalytic pathways (SOD like, CAT like, and GPX like) for both pristine Mn_3_O_4_ and the Mn_3_O_4_/SOD composite. For the SOD like pathway (conversion of O_2_·^−^ to H_2_O_2_), the rate determining step (RDS) energy barrier was calculated to be 0.54 eV for Mn_3_O_4_ NPs and 0.79 eV for the Mn_3_O_4_/SOD composite (Fig. 2j). This suggests that pristine Mn_3_O_4_ possesses superior intrinsic SOD like activity, potentially due to more accessible active sites. For the CAT like pathway (decomposition of H_2_O_2_ to H_2_O and O_2_), the RDS energy barrier was 0.94 eV for Mn_3_O_4_ and decreased to 0.84 eV for the Mn_3_O_4_/SOD composite (Fig. 2k). This lower barrier indicates that the presence of SOD enhances the CAT like activity, likely through synergistic interactions that facilitate H_2_O_2_ turnover. The GPX like pathway (reduction of H_2_O_2_ using GSH) exhibited the highest RDS energy barriers among the three activities: 1.95 eV for Mn_3_O_4_ and 1.51 eV for the composite (Fig. 2l). Although this pathway is kinetically less favorable, the presence of SOD substantially reduced the energy barrier by 0.44 eV, demonstrating a clear synergistic effect in enhancing GPX like catalytic efficiency.

In summary, the DFT calculations reveal that the Mn_3_O_4_/SOD hybrid exhibits enhanced CAT like and GPX like activities compared to Mn_3_O_4_ alone, underscoring a synergistic interplay between the nanozyme and the natural enzyme. This synergy is not merely additive but reflects a substrate channeling effect: the H_2_O_2_ generated by SOD is proximally available to Mn_3_O_4_, reducing diffusional loss and enabling a true cascade. The catalytic efficiency follows the order SOD like greater than CAT like greater than GPX like, which perfectly matches the kinetic demands of the APAP induced ROS burst (rapid O_2_·^−^ removal followed by H_2_O_2_ decomposition). These computational results provide a mechanistic foundation for the experimentally observed multifunctional antioxidant capacity of the SOD@ZIF-8/Mn_3_O_4_ nanocomposite (Figs. 2m-n).

In conclusion, we successfully fabricated a ternary SOD@ZIF-8/Mn_3_O_4_ nanocomposite through a step wise synthesis strategy. Comprehensive characterization confirmed its structural and compositional integrity. DFT calculations provided theoretical evidence for its multi enzyme mimetic activities (SOD, CAT, GPX) and revealed synergistic effects in the composite, highlighting its strong potential as a powerful antioxidant nanoplatform.

### Enzyme like antioxidant activity of SOD@ZIF-8/Mn_3_O_4_ nanoparticles

**Fig. 3:**
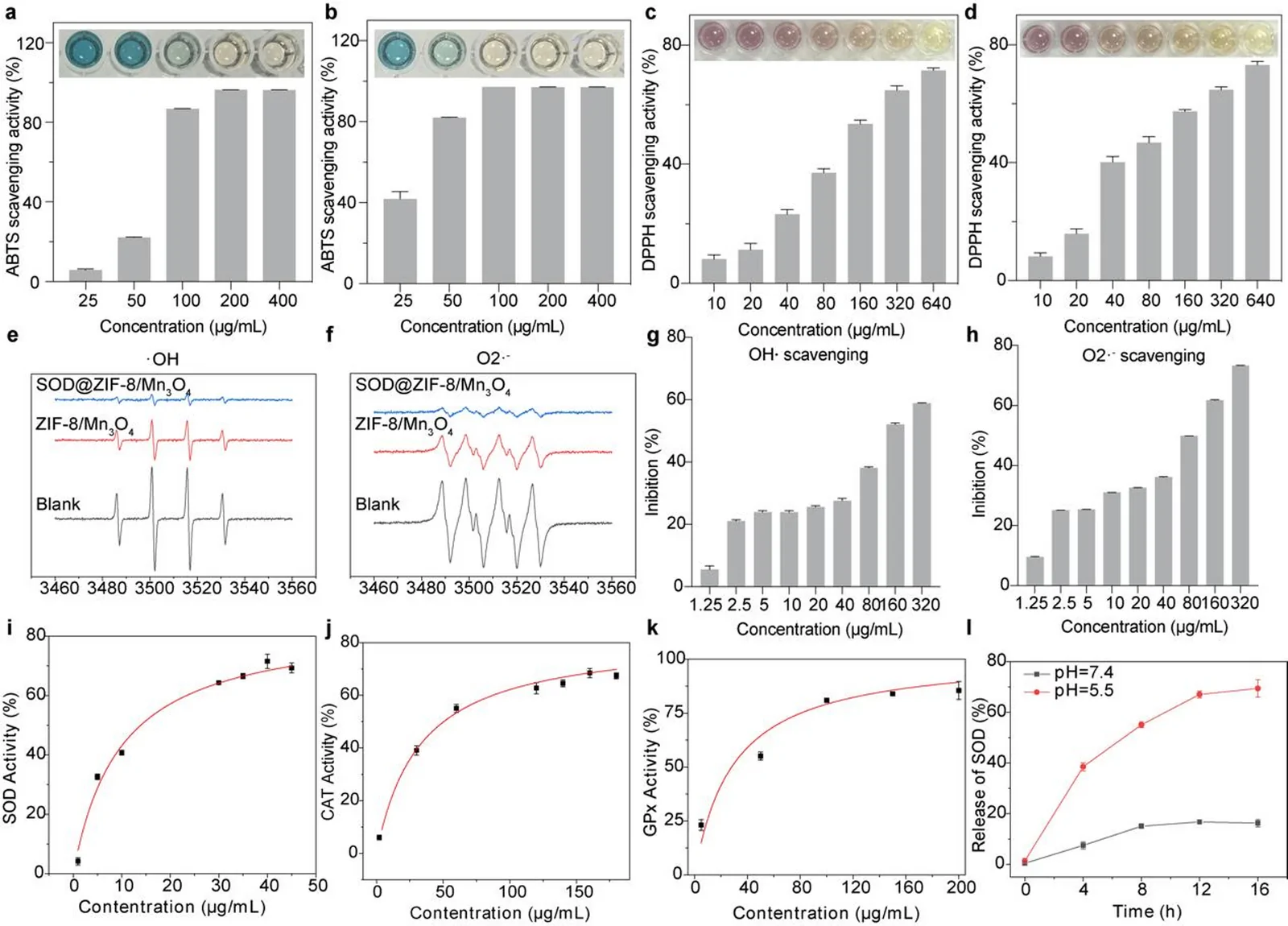
In vitro enzyme-mimetic and ROS-scavenging activities of SOD@ZIF-8/Mn_3_O_4_. (**a–d**) ABTS and DPPH radical scavenging activities of ZIF-8/Mn_3_O_4_ and SOD@ZIF-8/Mn_3_O_4_ at varying concentrations (*n* = 3). (**e–f**) EPR spectra of (**e**) ·OH and (**f**) O_2_·^−^ radicals in the presence of the nanocomposite. (**g–h**) Quantitative analysis of (**g**) ·OH and (**h**) O_2_·^−^scavenging efficiencies (*n* = 3). (**i–k**) Concentration-dependent (**i**) SOD-like, (**j**) CAT-like, and (**k**) GPX-like activities (*n* = 3), expressed as a percentage relative to the activity of 1 U/mL natural enzyme (defined as 100%). (**l**) Cumulative release profile of SOD from the nanocomposite at pH 7.4 and 5.5 (*n* = 3). Data are presented as mean ± SD, and the statistical significance was analyzed via one-way ANOVA. *P < 0.05, **P < 0.01, ***P < 0.001, and NS: Not significant

To evaluate the in vitro enzyme like catalytic performance of SOD@ZIF-8/Mn_3_O_4_, we assessed its ROS scavenging ability using colorimetric assays, which revealed potent ROS scavenging activity. As shown in Figs. 3a and b, both ZIF-8/Mn_3_O_4_ and SOD@ZIF-8/Mn_3_O_4_ were subjected to ABTS and DPPH assays. In the ABTS assay, the characteristic green coloration faded gradually as radical scavenging activity increased. SOD@ZIF-8/Mn_3_O_4_ exhibited significantly higher scavenging efficiency than ZIF-8/Mn_3_O_4_ across all tested concentrations. Notably, at concentrations ranging from 100 to 400 µg/mL, the scavenging efficiency of SOD@ZIF-8/Mn_3_O_4_ exceeded 80%, indicating its potent antioxidant activity. In the DPPH assay (Figs. 3c-d), color changes from purple to yellow reflected increasing scavenging activity. Again, SOD@ZIF-8/Mn_3_O_4_ consistently outperformed ZIF-8/Mn_3_O_4_ within the tested concentration range of 20 to 640 µg/mL, confirming its superior free radical neutralization capacity. As shown in Figure S4, the characteristic UV–vis absorption intensities of both ABTS and DPPH radicals decreased progressively with increasing nanoparticle concentrations, with SOD@ZIF-8/Mn_3_O_4_ exhibiting a more pronounced reduction than ZIF-8/Mn_3_O_4_. To further validate ROS scavenging activity at the molecular level, electron paramagnetic resonance (EPR) spectroscopy was employed (Figs. 3e-f).

At equivalent concentrations, SOD@ZIF-8/Mn_3_O_4_ demonstrated significantly lower signal intensities for both ·OH and superoxide anions O_2_·^-^, compared to ZIF-8/Mn_3_O_4_. These results strongly support its enhanced ROS scavenging performance. Relative quantitative analysis further revealed that at a concentration of 160 µg/mL, SOD@ZIF-8/Mn_3_O_4_ was capable of scavenging up to 60% of ·OH radicals (Fig. 3g). Under treatment conditions of 80 µg/mL, the scavenging rate of O_2_·^-^ exceeded 50% (Fig. 3h), confirming its substantial antioxidative potential. Collectively, these findings underscore the robust enzyme mimicking antioxidant capabilities of SOD@ZIF-8/Mn_3_O_4_, which outperforms its ZIF-8/Mn_3_O_4_ counterpart in both ABTS/DPPH radical assays and EPR based ROS scavenging evaluations. To further distinguish the CAT-like and GPx-like contributions of SOD@ZIF-8/Mn_3_O_4_, time-dependent H_2_O_2_ consumption was evaluated in the presence or absence of GSH. SOD@ZIF-8/Mn_3_O_4_ reduced residual H_2_O_2_ levels over time, and this effect was further enhanced by GSH, suggesting that the hybrid nanozyme could promote H_2_O_2_ detoxification through both CAT-like and GSH-assisted GPx-like catalytic pathways (Figure S5, supporting information). Moreover, time-dependent monitoring of O_2_·^-^, H_2_O_2_, and ·OH showed that the integrated SOD@ZIF-8/Mn_3_O_4_ nanocomposite achieved more efficient and sustained sequential ROS elimination than the physically mixed components, further supporting its cascade catalytic activity (Figure S6, supporting information).

SOD is a critical antioxidant enzyme. In this study, the WST-1 assay was utilized to evaluate the SOD like activity of the synthesized SOD@ZIF-8/Mn_3_O_4_ nanocomposite. As shown in Fig. 3i, the nanocomposite exhibited substantial enzymatic activity, achieving approximately 65% at a low concentration of 30 µg/mL. H_2_O_2_ is a ROS with greater membrane permeability and a longer lifetime than O_2_·^-^ and ·OH. The CAT like activity of SOD@ZIF-8/Mn_3_O_4_ was therefore investigated. The nanoparticles displayed concentration dependent CAT like activity, with decomposition efficiency exceeding 60% at 100 µg/mL (Fig. 3j). Additionally, the GPX like activity of the nanocomposite was evaluated, given its relevance to redox homeostasis via the GSH/GSSG cycle. Notably, the SOD@ZIF-8/Mn_3_O_4_ sample exhibited GPX like catalytic activity up to 80% at a concentration of 100 µg/mL (Fig. 3k), indicating broad spectrum antioxidant capabilities. For each enzyme like activity assay, a standard curve was generated using serial dilutions of the corresponding natural enzyme (SOD, CAT, or GPx) with known specific activities. The activity of the nanocomposite was interpolated from the standard curve and expressed as a percentage relative to the activity of 1 U/mL of the natural enzyme, which was defined as 100%. To quantitatively evaluate the catalytic efficiency of SOD@ZIF-8/Mn_3_O_4_ toward its primary substrate O_2_·^-^, we performed a kinetic analysis using the WST-1 method. The initial reaction velocities were measured at varying initial substrate concentrations, and the data were fitted to the Michaelis-Menten equation. As illustrated in Figure S7 (Supporting information), the reaction velocity exhibited a typical hyperbolic dependence on substrate concentration, characteristic of Michaelis-Menten kinetics. Nonlinear regression analysis yielded a maximum reaction velocity (Vmax) of 0.28 ± 0.09 µM/s and a Michaelis constant (Km) of 7.15 ± 1.46 mM for SOD@ZIF-8/Mn_3_O_4_. (Figure S3b, Supporting information). The relatively low Km value indicates that the nanocomposite possesses high affinity toward superoxide anions, while the Vmax value reflects its efficient catalytic turnover. These kinetic parameters demonstrate that SOD@ZIF-8/Mn_3_O_4_ exhibits excellent catalytic efficiency toward O_2_·^-^, which mechanistically underpins the potent ROS scavenging and cytoprotective effects observed in our cellular and in vivo studies. In addition, the CAT-like and GPx-like catalytic kinetics of SOD@ZIF-8/Mn_3_O_4_ were further evaluated, yielding Km values of 12.45 ± 0.59 mM and 3.52 ± 0.08 mM, respectively, and corresponding Vmax values of 0.17 ± 0.04 µM/s and 0.09 ± 0.01 µM/s, as shown in Figure S7 (Supporting Information).

To assess the release of encapsulated SOD under physiological pH conditions, in vitro release studies were performed at pH 7.4 and pH 5.5. As shown in Fig. 3l, SOD@ZIF-8/Mn_3_O_4_ remained stable at physiological pH 7.4, while demonstrating a controlled, gradual dissociation in acidic conditions (pH 5.5), mimicking the intracellular environment of inflamed tissues. To further confirm the successful encapsulation of SOD, FITC labeled SOD@ZIF-8/Mn_3_O_4_ was analyzed using confocal laser scanning microscopy with Z stack imaging. As illustrated in Figure S8 (Supporting information), the fluorescence signal exhibited a gradual increase followed by a decrease across the optical sections, suggesting that SOD was embedded within the interior of the ZIF-8 rather than being adsorbed onto its surface.

Collectively, these findings confirm that SOD@ZIF-8/Mn_3_O_4_ exhibits efficient enzyme mimicking activity against multiple ROS targets, specifically ABTS, DPPH, ·OH, and O_2_·^-^, while also enabling pH responsive release of biologically active SOD. This multifunctional capability underscores its potential as a robust antioxidative therapeutic nanoplatform. Importantly, the pH responsive release profile (Fig. 3l) ensures that SOD is mainly liberated within the acidic endosomal or lysosomal compartments and the inflamed liver microenvironment, minimizing premature loss of activity. This is a critical advantage over free SOD.

### Cellular uptake and intracellular release of SOD@ZIF-8/Mn_3_O_4_

**Fig. 4:**
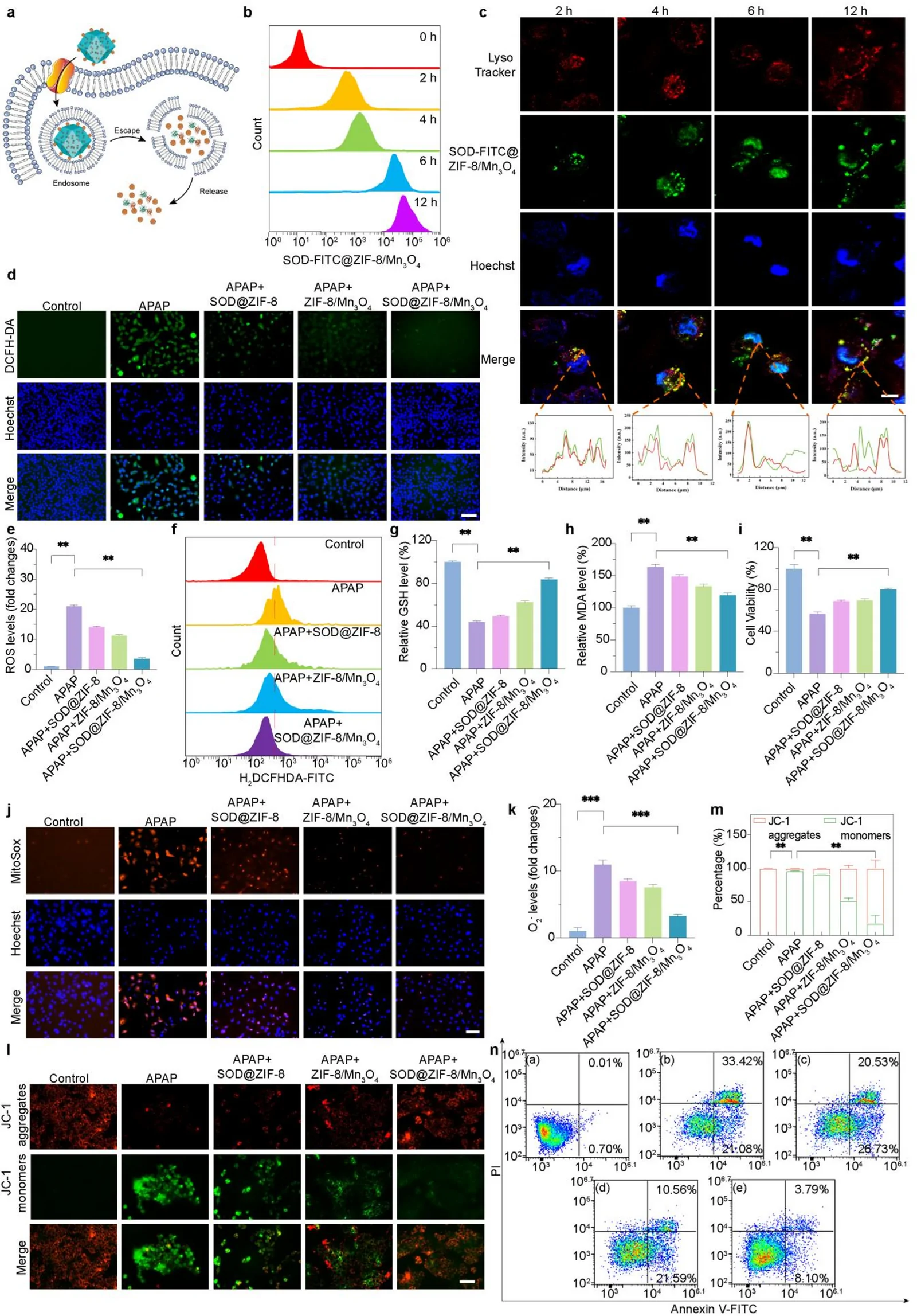
Cellular uptake and cytoprotective effects of SOD@ZIF-8/Mn_3_O_4_ in APAP-injured AML-12 hepatocytes. (**a**) Schematic of cellular internalization and lysosomal escape. (**b**) Flow cytometry analysis of cellular uptake over time. (**c**) Confocal images showing lysosomal escape of FITC-labeled nanocomposite (green) over 12 h. Lysotracker Red stains lysosomes; scale bar: 10 μm. (d–f) Intracellular ROS levels detected by DCFH-DA: (**d**) fluorescence images (scale bar: 200 μm), (**e**) quantitative analysis, and (**f**) flow cytometry histograms. (**g–h**) Levels of (**g**) glutathione (GSH) and (**h**) malondialdehyde (MDA) (*n* = 3). (**i**) Cell viability assessed by CCK-8 assay (*n* = 5). (**j–k**) Detection of O_2_·^⁻^ in AML-12 cells under different conditions using MitoSox probe. (**j**) Fluorescent micrographs. Scale bars: 200 μm. (k) Relative quantification data (*n* = 3). (**l–m**) Mitochondrial membrane potential assessed by JC-1 staining: (**l**) images. Scale bar: 200 μm. and (**m**) red/green fluorescence ratio (*n* = 3). (**n**) Apoptosis analysis by flow cytometry. Data are presented as mean ± SD, and the statistical significance was analyzed via one-way ANOVA. *P < 0.05, **P < 0.01, ***P < 0.001, and NS: not significant

The above results indicate that the rational co assembly of SOD and Mn_3_O_4_ within ZIF-8 NPs facilitates the construction of a nanozyme system with integrated multi enzyme mimetic activities. To investigate the potential effects of SOD@ZIF-8/Mn_3_O_4_ on normal mouse hepatocytes, its cytotoxicity was initially evaluated in normal murine hepatocyte AML-12 cells. Initially, AML-12 cells were incubated with varying concentrations of SOD or Mn_3_O_4_ nanoparticles for 24 h. As shown in Figure S9 and S10 (Supporting information), cell viability maintained above 80% at different tested concentrations, indicating low toxicity and favorable biocompatibility of the developed nanomaterials. Subsequently, the intracellular uptake and distribution of SOD@ZIF-8/Mn_3_O_4_ (Fig. 4a) were evaluated using CLSM and flow cytometry. As illustrated in Figure S11 (Supporting information), time dependent increases in intracellular fluorescence were observed, with prominent green fluorescence localized around the nucleus after 12 h of incubation. These results suggest efficient cellular internalization of the nanocomposite. Correspondingly, flow cytometric analysis (Fig. 4b) showed a progressive increase in the proportion of fluorescent positive AML-12 cells over time, confirming effective endocytosis and cytoplasmic delivery. These results suggest that SOD@ZIF-8/Mn_3_O_4_ possesses favorable biocompatibility and can be effectively internalized by AML-12 cells. Subsequently, the capability of the nanoparticles to escape from lysosomes was systematically evaluated. The ability of SOD@ZIF-8/Mn_3_O_4_ to escape lysosomes was verified using confocal microscopy and fluorescence co localization analysis (Fig. 4c). At early time points (2 to 4 h), green (FITC) and red (Lysotracker) fluorescence signals overlapped, indicating nanoparticle entrapment within lysosomes. By 6 h, signal separation began. By 12 h, complete dissociation of the signals was observed, confirming successful lysosomal escape which is critical for cytoplasmic delivery of the nanoparticles.

### SOD@ZIF-8/Mn_3_O_4_ mitigates intracellular oxidative stress via cascade catalytic activity

To simulate ALI in vitro, AML-12 cells were exposed to different concentrations of APAP, and cell viability was measured after 24 h. As shown in Figure S12 (Supporting information ), IC50 was determined to be 1.2 mg/mL, which was subsequently used in all APAP induced injury experiments in vitro. To determine the optimal working concentration for subsequent cellular experiments, the cytoprotective effects of different concentrations of SOD@ZIF-8/Mn_3_O_4_ were evaluated in APAP-injured AML-12 cells using the CCK-8 assay. As shown in Figure S13, SOD@ZIF-8/Mn_3_O_4_ restored cell viability in a concentration-dependent manner, and 10 µg/mL was the lowest tested concentration that produced a significant protective effect. Therefore, 10 µg/mL was selected as the working concentration for the subsequent in vitro experiments. To quantitatively assess the synergistic interaction between SOD and Mn_3_O_4_, Combination Index (CI ) analysis was performed based on the fraction affected (Fa ). As shown in Figure S14, at a Fa of 0.5, the CI value was found to be less than 1 indicating a synergistic rather than merely additive interaction between the SOD-containing and Mn_3_O_4_-containing components. This result supports the hierarchical catalyst design of SOD@ZIF-8/Mn_3_O_4_, in which SOD-mediated O_2_·^-^ dismutation is coupled with Mn_3_O_4_-mediated H_2_O_2_ decomposition and ·OH suppression, thereby achieving efficient cascade ROS detoxification. This synergy is mechanistically attributable to the catalytic hierarchy wherein SOD converts O_2_·^-^ to H_2_O_2_, and Mn_3_O_4_ immediately decomposes H_2_O_2_, preventing the Fenton reaction that would otherwise generate highly toxic ·OH. Such a two step relay is impossible with either component alone, and the ZIF-8 scaffold ensures spatial proximity. APAP induced liver injury is strongly associated with the generation of ROS. The protective effect of SOD against APAP induced hepatocellular damage was evaluated at the cellular level using the DCFDA probe. In contrast, cells pretreated with SOD@ZIF-8/Mn_3_O_4_ showed a substantial reduction in ROS accumulation approximately sixfold lower, effectively restoring redox balance within the cells (Fig. 4d-e ). Flow cytometry further corroborated these findings. As shown in Fig. 4f, the APAP treated group exhibited a significant rightward shift, whereas treatment with SOD@ZIF-8/Mn_3_O_4_ nearly completely reversed the oxidative damage. Its ROS scavenging capacity was markedly higher than that of the other groups (SOD@ZIF-8 and ZIF-8/Mn_3_O_4_ ), further validating the synergistic cascade catalytic effect. Excessive administration of APAP leads to the depletion of intracellular glutathione (GSH ), thereby inducing lipid peroxidation and increasing malondialdehyde (MDA ) production. To further elucidate the in vitro antioxidant capacity of SOD, intracellular levels of GSH and MDA were quantitatively assessed. In the APAP treated group, a marked reduction in GSH was observed, accompanied by a significant increase in MDA levels. Conversely, treatment with SOD@ZIF-8/Mn_3_O_4_ markedly restored GSH content and decreased MDA accumulation, effectively alleviating oxidative stress at the cellular level. Notably, this antioxidative effect surpassed that of both the SOD@ZIF-8 and ZIF-8/Mn_3_O_4_ groups, highlighting a synergistic cascade catalytic mechanism (Fig. 4g-h ).

To investigate the protective effects of SOD@ZIF-8/Mn_3_O_4_ on APAP induced hepatocyte injury, a series of assays were subsequently performed. The cytoprotective effect of SOD@ZIF-8/Mn_3_O_4_ was first evaluated using the CCK-8 assay. As shown in Fig. 4i, pretreatment with SOD@ZIF-8/Mn_3_O_4_ significantly restored cell viability in APAP injured AML-12 cells, with superior efficacy compared to SOD@ZIF-8 and ZIF-8/Mn_3_O_4_ alone. This indicates that the nanocomposite exerts synergistic protective effects against APAP induced cytotoxicity. Live and Dead staining showed that APAP exposure significantly increased red fluorescence (dead cells) and reduced green fluorescence (live cells) in AML-12 cells (Figure S15, Supporting information). Pretreatment with SOD@ZIF-8/Mn_3_O_4_ reversed this effect, markedly reducing red fluorescence and increasing green fluorescence. These results collectively demonstrate that SOD@ZIF-8/Mn_3_O_4_ can reduce the excessive accumulation of intracellular ROS caused by APAP, decrease lipid peroxidation levels, restore cell viability, and reduce cell death. Moreover, SOD and Mn_3_O_4_ exhibit a synergistic cascade catalytic effect.

### SOD@ZIF-8/Mn_3_O_4_ effectively reverses intracellular lipid peroxidation

These results prompted further investigation of the effect of SOD@ZIF-8/Mn_3_O_4_ on intracellular lipid peroxidation. BODIPY 581/591 C11 is a sensitive fluorescent dye commonly used for detecting lipid peroxidation in cells. During redox reactions, it undergoes a fluorescence shift from red (reduced state) to green (oxidized state), indicating the degree of lipid peroxidation. As shown in Figure S16 (Supporting information), APAP treatment led to an increase in green fluorescence, indicating a shift toward the oxidized state of the probe. In contrast, the SOD@ZIF-8/Mn_3_O_4_ pretreated group showed a marked restoration of red fluorescence, with better results than the other two groups (SOD@ZIF-8 and ZIF-8/Mn_3_O_4_), suggesting a synergistic anti lipid peroxidation effect.

To directly assess the scavenging capacity of SOD@ZIF-8/Mn_3_O_4_ toward its primary substrate O_2_·^-^, we measured intracellular O_2_·^-^ levels using MitoSOX Red fluorescent probes in APAP injured AML-12 cells. As shown in Figs. 4j-k, APAP treatment significantly increased fluorescence intensity by approximately 10.9 fold compared to control cells, indicating marked accumulation of total intracellular O_2_·^-^. Pretreatment with SOD@ZIF-8/Mn_3_O_4_ substantially reduced fluorescence by 70.25% compared to the APAP alone group, demonstrating potent total O_2_·^-^ scavenging activity. These results provide direct evidence that SOD@ZIF-8/Mn_3_O_4_ effectively targets and neutralizes total cellular O_2_·^-^, validating the proposed cascade catalytic mechanism and supporting its role as a highly efficient superoxide scavenging nanozyme. Lipid peroxidation impairs the mitochondrial membrane, leading to a decrease in mitochondrial membrane potential (Δψm). JC-1 is a well established fluorescent probe used to detect Δψm. In healthy mitochondria, JC-1 aggregates and emits red fluorescence. Changes in the red/green fluorescence intensity ratio can be used for semi quantitative assessment of mitochondrial membrane potential. As shown in Figs. 4l-m, APAP treatment significantly reduced red fluorescence, indicating that JC-1 remained in its monomeric form due to Δψm loss. Following SOD@ZIF-8/Mn_3_O_4_ treatment, the red/green fluorescence ratio increased, suggesting a restoration of mitochondrial membrane potential. Notably, this recovery was more pronounced than that observed with either SOD@ZIF-8 or ZIF-8/Mn_3_O_4_ alone, highlighting the synergistic cascade effect of the combined system in reversing APAP induced mitochondrial dysfunction.

Following mitochondrial damage, increased mitochondrial membrane permeability leads to the initiation of programmed cell death. To further visually verify the protective effect of SOD@ZIF-8/Mn_3_O_4_ on APAP induced cells, we conducted a flow cytometry assay to assess apoptosis. Quantitative analysis showed that the apoptotic rate decreased from 54.50% in the APAP group to just 11.89% in the SOD@ZIF-8/Mn_3_O_4_ group, outperforming SOD@ZIF-8 (47.26%) and ZIF-8/Mn_3_O_4_ (32.15%) (Fig. 4n and Figure S17, Supporting information).

In APAP induced ALI, increased apoptosis and impaired proliferation jointly contribute to the progression of hepatocellular damage. Meanwhile, cellular proliferation levels were also assessed. As shown in Figure S18 (Supporting information), APAP treatment markedly suppressed cell proliferation, as evidenced by a significant reduction in EdU fluorescence intensity. However, pre treatment with SOD@ZIF-8/Mn_3_O_4_ significantly attenuated this effect, resulting in nearly a two fold increase in fluorescence intensity compared to the APAP group, indicating a protective effect on cellular proliferation capacity.

When cells are exposed to an overdose of APAP, a large amount of ROS, particularly H_2_O_2_ and O_2_·^-^, is generated in hepatocytes. This leads to the depletion of intracellular GSH, inhibition of GPX4 activity, and ultimately triggers lipid peroxidation [42, 43]. This mechanism plays a critical role in liver pathologies and represents a promising target for therapeutic intervention. APAP exposure resulted in the downregulation of several lipid peroxidation regulating proteins, including GPX4, SLC7A11, and ferroptosis suppressor protein-1 (FSP-1) (Figure S19, Supporting information). SLC7A11 facilitates cystine uptake for GSH synthesis, while FSP-1 operates via a GPX4 independent mechanism to suppress lipid peroxidation. Western blot analysis confirmed that SOD@ZIF-8/Mn_3_O_4_ restored the expression levels of these proteins to near normal levels. These results collectively demonstrate that SOD@ZIF-8/Mn_3_O_4_ effectively protects hepatocytes from lipid peroxidation by reducing ROS. The restoration of GPX4, SLC7A11 and FSP-1 indicates that the nanozyme intervenes at an early stage of the ferroptosis pathway, preventing the commitment to cell death. This dual action antioxidant and anti lipid peroxidation mechanism highlights its therapeutic potential for the prevention and treatment of oxidative stress related liver injuries, including APAP induced hepatotoxicity.

### Molecular mechanism of the antioxidant effect of SOD@ZIF-8/Mn_3_O_4_

**Fig. 5:**
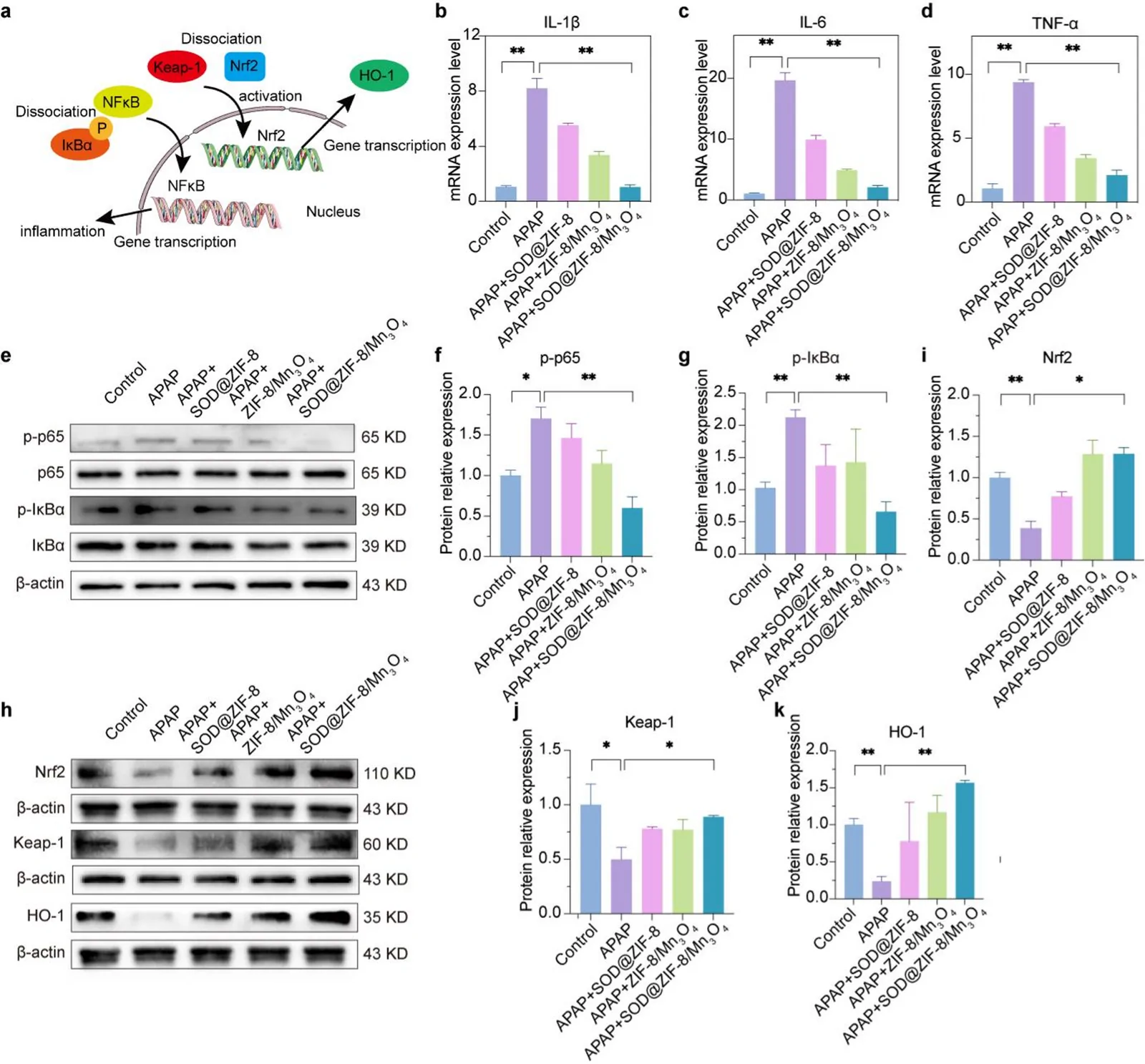
Molecular mechanisms underlying the antioxidant and anti-inflammatory effects of SOD@ZIF-8/Mn_3_O_4_ in AML-12 cells. (**a**) Schematic of the Keap1/Nrf2/HO-1 and NF-κB pathways. (**b–d**) qPCR analysis of pro-inflammatory cytokines: (**b**) IL-1β, (**c**) IL-6, and (**d**) TNF-α. (**e–g**) Western blot analysis and quantification of Keap1, Nrf2, and HO-1 expression. (**h–k**) Western blot and quantification of p-IκBα, IκBα, p-p65, and p65. All experiments were performed in triplicate. Data are presented as mean ± SD, and the statistical significance was analyzed via one-way ANOVA. *P < 0.05, **P < 0.01, ***P < 0.001, and NS: Not significant

To elucidate the underlying molecular mechanism of the antioxidant activity of SOD@ZIF-8/Mn_3_O_4_, Western blot analysis was performed to assess the expression and activation of key signaling molecules involved in oxidative stress regulation and inflammation in APAP treated AML-12 cells. Under normal conditions, the transcription factor Nrf2 is sequestered in the cytoplasm by Keap1, preventing its activation. Oxidative stress induced by APAP disrupts this complex, leading to Nrf2 translocation into the nucleus. The expression of NRF2 declined, resulting in downregulation of its downstream effector, HO-1 (Fig. 5a). WB results revealed that APAP declined Keap1 expression while impairing Nrf2 mediated cytoprotective signaling. However, treatment with SOD@ZIF-8/Mn_3_O_4_ reactivated the Keap1/Nrf2/HO-1 axis, as evidenced by the increased expression of Keap1 and Nrf2 and HO-1 upregulation. This enhanced the cellular defence response against oxidative stress. APAP induced oxidative stress was also found to activate the NF-κB pathway. This was characterized by phosphorylation of IκBα and NF-κB, and subsequent transcriptional upregulation of pro inflammatory cytokines including IL-1β, IL-6, and TNF-α (Figs. 5b-d). Notably, SOD@ZIF-8/Mn_3_O_4_ treatment effectively reversed NF-κB and IκBα phosphorylation and significantly suppressed the expression of these inflammatory mediators. This inhibitory effect was more pronounced than that observed with either SOD@ZIF-8 or ZIF-8/Mn_3_O_4_ alone, suggesting a synergistic anti inflammatory effect. SOD@ZIF-8/Mn_3_O_4_ exerts its antioxidant and anti inflammatory effects through dual modulation of two critical pathways. First, it activates the Keap1/Nrf2/HO-1 axis, thereby enhancing the cellular antioxidant response (Figs. 5e-g). It also inhibits the NF-κB signalling cascade, thereby suppressing the expression of inflammatory genes (Figs. 5h-k). This dual regulatory mechanism underscores the therapeutic potential of SOD@ZIF-8/Mn_3_O_4_ in managing oxidative stress and inflammation associated with APAP induced hepatotoxicity. Notably, rather than simply upregulating Nrf2, the nanozyme restored its interaction with Keap1 (evidenced by normalized Keap1 expression), suggesting re establishment of the redox sensitive “off switch”. This feature is rarely achieved by small molecule antioxidants. Meanwhile, NF-κB suppression occurred downstream of ROS reduction, indicating that the nanozyme breaks the ROS inflammation feed forward loop, a key pathological driver of ALI progression.

To validate whether the Nrf2/HO-1 and NF-κB pathways mediate the protective effects of SOD@ZIF-8/Mn_3_O_4_, inhibitor based experiments were conducted. As shown in the Figure S20 (Supporting information), CCK-8 assays revealed that APAP treatment significantly reduced cell viability to 52.89% of control levels, whereas SOD@ZIF-8/Mn_3_O_4_ treatment restored viability to 79.64%. However, co treatment with ML385 partially reversed this protective effect, reducing cell viability to 65.37%, which was significantly different from the APAP plus SOD@ZIF-8/Mn_3_O_4_ group. ROS measurements were consistent with the cell viability data. SOD@ZIF-8/Mn_3_O_4_ significantly reduced APAP induced ROS levels, whereas ML385 co treatment markedly diminished this scavenging effect. Specifically, ROS levels increased again upon ML385 addition, indicating that ML385 partially abrogated the ROS scavenging activity of SOD@ZIF-8/Mn_3_O_4_. These results confirm that the protective effect of SOD@ZIF-8/Mn_3_O_4_ against APAP induced injury is dependent on Nrf2 pathway activation. To further assess the role of NF-κB signaling in the anti inflammatory effects of SOD@ZIF-8/Mn_3_O_4_, the NF-κB inhibitor BAY 11–7082 was used. CCK-8 results showed that BAY 11–7082 treatment restored cell viability to 71.83% in APAP injured AML-12 cells, and also reduced ROS levels. Notably, co treatment with BAY 11–7082 and SOD@ZIF-8/Mn_3_O_4_ further enhanced the antioxidant effect of SOD@ZIF-8/Mn_3_O_4_, leading to decreased ROS levels and increased cell proliferation, suggesting that SOD@ZIF-8/Mn_3_O_4_ alleviates APAP induced inflammation by inhibiting NF-κB pathway activation.

### Anti-acute liver injury effects of SOD@ZIF-8/Mn_3_O_4_ NPs in vivo

**Fig. 6:**
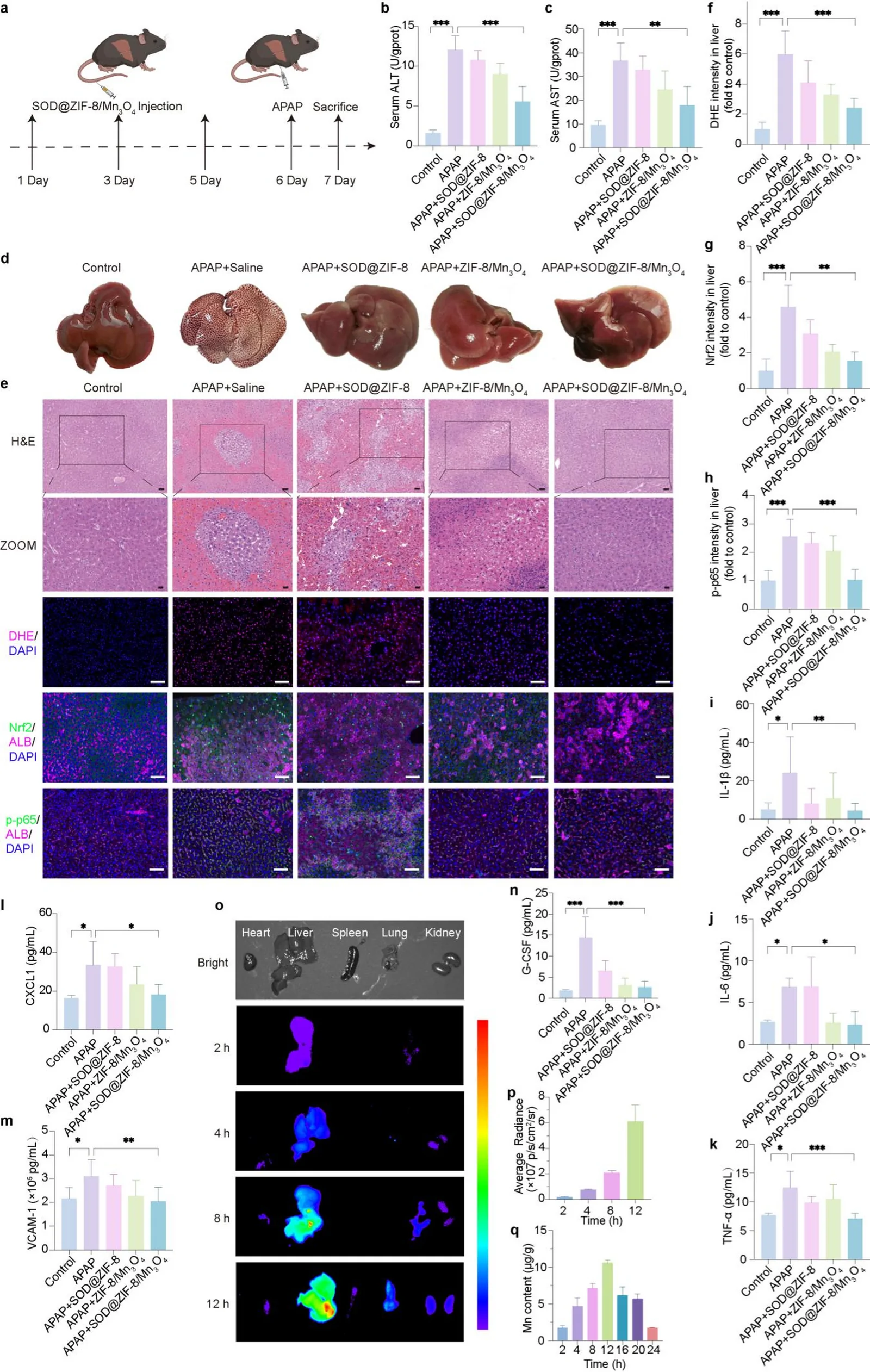
In vivo therapeutic efficacy of SOD@ZIF-8/Mn_3_O_4_ in a murine model of APAP-induced ALI. (**a**) Experimental timeline (*n* = 7). (**b–c**) Serum levels of (**b**) ALT and (**c**) AST. (**d**) Macroscopic appearance of liver tissues. (**e**) H&E staining and immunofluorescence staining of liver sections (scale bar: 50 μm). (**f–h**) Quantification of immunofluorescence signals for Nrf2 and p-p65 (*n* = 5). (**i–m**) Serum levels of inflammatory mediators: (**i**) IL-1β, (**j**) IL-6, (**k**) TNF-α, (**l**) CXCL1, (**m**) VCAM-1 and (**n**) G-CSF. (**o–p**) In vivo fluorescence imaging and quantification of liver accumulation over time (*n* = 3). (**q**) Biodistribution of Mn in major organs measured by ICP-MS (*n* = 3). Data are presented as mean ± SD, and the statistical significance was analyzed via one-way ANOVA. *P < 0.05, **P < 0.01, ***P < 0.001, and NS: Not significant

Given the demonstrated ROS scavenging activity, antioxidant enzyme mimicry, lipid peroxidation inhibition, and favorable biodistribution of SOD@ZIF-8/Mn_3_O_4_ in vitro, we next assessed their therapeutic potential in a murine model of APAP induced ALI. The model mice were intravenously pretreated with SOD@ZIF-8, ZIF-8/Mn_3_O_4_ and SOD@ZIF-8/Mn_3_O_4_ three times prior to APAP challenge (Fig. 6a). 24 h post APAP administration, serum levels of alanine aminotransferase (ALT) and aspartate aminotransferase (AST) were measured. These are biochemical markers of ALI. As expected, APAP exposure led to a significant elevation of both ALT and AST levels (Figs. 6b-c), confirming successful model induction. In contrast, SOD@ZIF-8/Mn_3_O_4_ pretreated groups exhibited markedly reduced ALT and AST levels, indicating effective hepatoprotection. The livers of APAP treated mice exhibited a whitish hue, diminished blood supply, and a rough texture, which are characteristic of hepatic necrosis. In contrast, SOD@ZIF-8/Mn_3_O_4_ treated livers displayed a return to healthy pink coloration and smooth surfaces (Fig. 6d). H&E staining of liver sections (Fig. 6e) demonstrated severe hepatocellular necrosis in the APAP group. These pathological features were significantly alleviated in SOD@ZIF-8/Mn_3_O_4_ pretreated mice, supporting the hepatoprotective capacity of SOD@ZIF-8/Mn_3_O_4_.

As shown in Fig. 6e, a considerable fluorescence intensity of DHE was observed in the APAP group, which was much weaker in the group pretreated with SOD@ZIF-8/Mn_3_O_4_. From the immunofluorescence staining images, it can be observed that APAP induced pronounced nuclear translocation of Nrf2 in hepatocytes (marked by ALB), indicating the occurrence of oxidative stress. In addition, hepatocytes exhibited elevated expression of p-p65, further confirming the oxidative stress status in the liver. In contrast, SOD@ZIF-8/Mn_3_O_4_ markedly alleviated the oxidative stress caused by APAP, demonstrating a strong protective effect on hepatocytes and reducing oxidative damage in these cells (Figs. 6f-h). This result corroborates the proposed mechanism by which SOD@ZIF-8/Mn_3_O_4_ mitigates both oxidative stress.

APAP overdose is known to trigger innate immune responses by activating resident hepatic macrophages (Kupffer cells), which release pro inflammatory cytokines and chemokines. These include IL-1β, IL-6, TNF-α and CXCL1, which promote neutrophil recruitment and exacerbate hepatic inflammation. Additionally, granulocyte colony stimulating factor (G-CSF) and vascular cell adhesion molecule-1 (VCAM-1) are secreted to enhance immune cell mobilization and adhesion, further exacerbating liver injury. To evaluate the anti inflammatory effect of the SOD@ZIF-8/Mn_3_O_4_ nanoplatform, serum levels of these inflammatory mediators were measured post treatment (Figs. 6i–n). The APAP model group exhibited significantly elevated levels of all five markers, confirming the presence of acute liver inflammation. Notably, pretreatment with SOD@ZIF-8/Mn_3_O_4_ markedly reduced the serum concentrations of IL-1β, IL-6, TNF-α, CXCL1, G-CSF, and VCAM-1. These reductions were significantly greater than those observed in the SOD@ZIF-8 and ZIF-8/Mn_3_O_4_ control groups, highlighting the superior anti inflammatory effect of the dual functional composite.

To confirm organ specific targeting and accumulation, in vivo fluorescence imaging was conducted following intravenous administration of SOD@ZIF-8/Mn_3_O_4_ in APAP induced liver injury mice. Time course imaging revealed a progressive increase in liver localized fluorescence signal, peaking at approximately 12 h post injection. This biodistribution pattern clearly demonstrates the preferential hepatic accumulation of SOD@ZIF-8/Mn_3_O_4_, supporting its targeted therapeutic effect in liver injury (Figs. 6o-p). As shown in Fig. 6q, the Mn content in liver tissues was quantified by inductively coupled plasma mass spectrometry (ICP-MS). The results showed that Mn accumulation in the liver peaked at 12 h, which was consistent with the fluorescence imaging results. Thereafter, the Mn level gradually decreased and reached its lowest level at 24 h, suggesting time-dependent hepatic clearance of the Mn-containing nanocomposite. Remarkably, SOD@ZIF-8/Mn_3_O_4_ achieved therapeutic efficacy comparable to the first line drug NAC (Figure S21, Supporting information), as evidenced by equivalent reductions in ALT/AST and histopathological recovery. However, unlike NAC, which acts mainly as a glutathione precursor and has a narrow therapeutic window, our nanozyme provides sustained, self catalytic ROS elimination and exhibits liver specific accumulation, offering a potential advantage for delayed intervention.

### Action mechanism of SOD@ZIF-8/Mn_3_O_4_ NPs on ALI mice

**Fig. 7:**
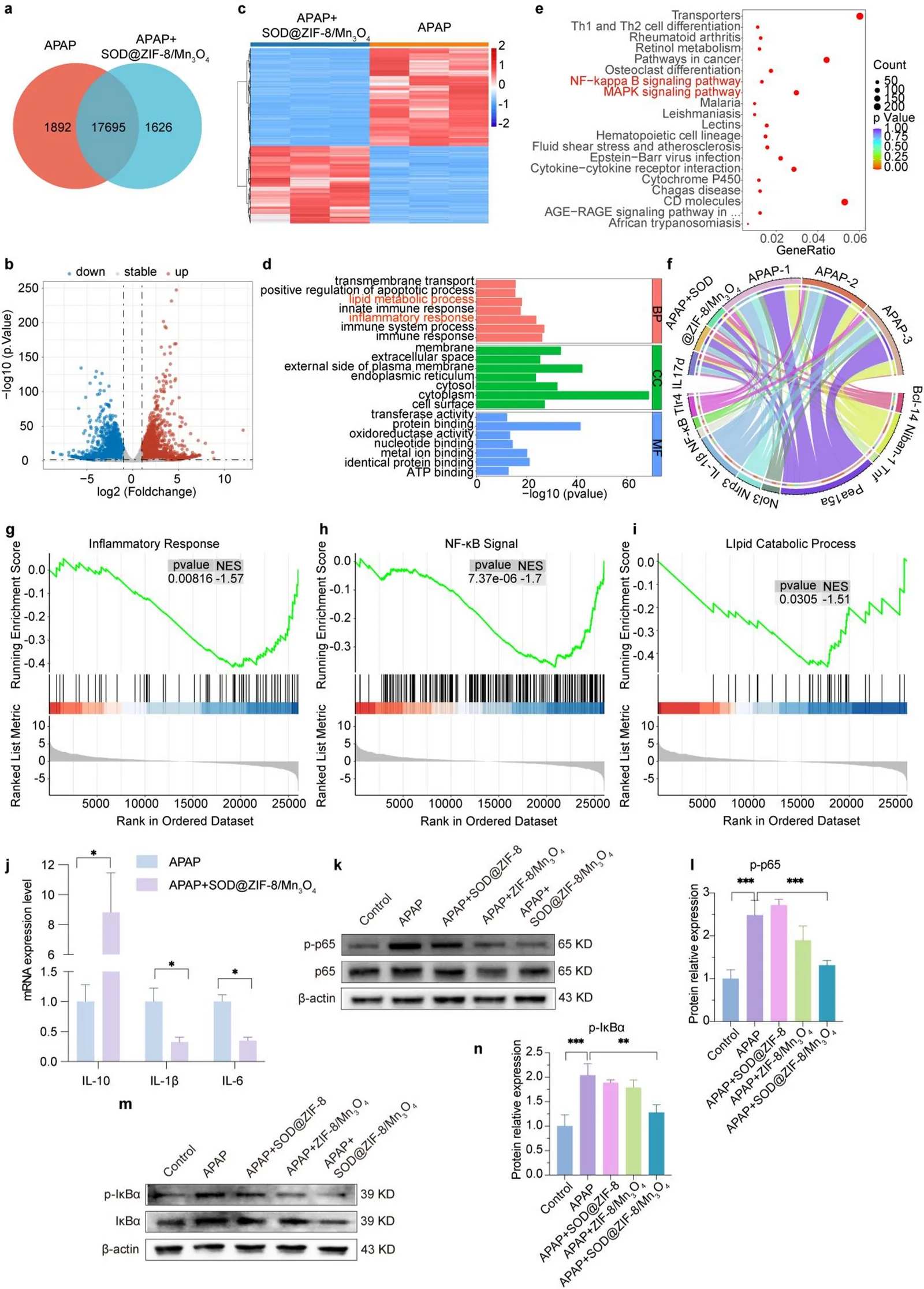
Therapeutic mechanisms of SOD@ZIF-8/Mn_3_O_4_ on ALI in vivo. (**a**) Venn diagram of the transcriptomic profiles between the APAP group and the APAP + SOD@ZIF-8/Mn_3_O_4_ group. (**b**) Volcano plot of the significantly upregulated and downregulated genes after SOD@ZIF-8/Mn_3_O_4_ treatment. (**c**) Heatmap of significantly differentially expressed genes. (**d**) GO enrichment analysis (BP, CC, and MF stand for biological process, cellular components, and molecular function). (**e**) The top 20 significantly enriched KEGG pathways. (**f**) Circos analysis of relevant genes. (**g**)-(**i**) Gene set enrichment analysis (GSEA). (**j**) Relative expression levels of inflammatory cytokines. Western blot (**k**), (**m**) and relative protein expression analysis (**l**), (**n**) (*n* = 3). Data are presented as mean ± SD, and the statistical significance was analyzed via one-way ANOVA. *P < 0.05, **P < 0.01, ***P < 0.001, and NS: Not significant

To further elucidate the molecular mechanisms underlying the therapeutic effect of SOD@ZIF-8/Mn_3_O_4_ in ALI, transcriptome sequencing was performed on liver tissues collected from mice treated with APAP alone and those treated with APAP plus SOD@ZIF-8/Mn_3_O_4_. Venn diagram analysis (Fig. 7a) revealed a total of 17,695 genes expressed across both groups, with 1,626 genes uniquely expressed in the SOD@ZIF-8/Mn_3_O_4_ treated group, suggesting a significant transcriptional impact of the nanotherapy. Volcano plot analysis (Fig. 7b) identified a substantial number of differentially expressed genes (DEGs). A heatmap (Fig. 7c) provided further visualization of the gene expression patterns, highlighting distinct transcriptional differences between treatment and control groups. Gene Ontology (GO) and Kyoto Encyclopedia of Genes and Genomes (KEGG) enrichment analyses were performed on the DEGs to identify the key biological processes and pathways affected by SOD@ZIF-8/Mn_3_O_4_ (Figs. 7d-e). Enriched pathways included those involved in transporters, Th1 and Th2 cell differentiation, NF-kappa B signaling pathway, MAPK signaling pathway and so on. GO enrichment analysis further revealed that biological processes (BP) related to lipid metabolism were significantly enriched, suggesting a strong association with lipid peroxidation.

This enrichment supports the hypothesis that SOD@ZIF-8/Mn_3_O_4_ mitigates APAP induced liver injury in part by regulating lipid metabolic pathways, thereby inhibiting lipid peroxidation. GO enrichment analysis further revealed that BP related to lipid metabolism were significantly enriched, suggesting a strong association with lipid peroxidation. These results corroborate previous in vitro and in vivo findings (Figs. 5 and 6), confirming that SOD@ZIF-8/Mn_3_O_4_ exerts its protective effects via a dual mechanism: attenuating inflammatory responses.

Circos analysis identified differentially expressed genes associated with oxidative stress, inflammation, and apoptosis (Fig. 7f). Gene set enrichment analysis further validated the involvement of key regulatory pathways, with enriched gene sets significantly associated with inflammatory responses, NF-κB signaling, and lipid catabolic processes (Figs. 7g–i). Consistently, SOD@ZIF-8/Mn_3_O_4_ treatment reduced the mRNA expression of inflammatory factors (Fig. 7j). Western blot analysis showed that APAP treated liver tissues exhibited marked phosphorylation of p65 and IκBα, indicating NF-κB pathway activation; this phosphorylation was significantly suppressed by SOD@ZIF-8/Mn_3_O_4_ (Fig. 7k and m). Furthermore, immunofluorescence staining was performed to evaluate GPX4 expression in liver tissues from the different treatment groups (Figure S22, Supporting Information). To further evaluate its therapeutic efficacy rather than its preventive effect, SOD@ZIF-8/Mn_3_O_4_ was administered after APAP-induced liver injury was established. Compared with the APAP group, therapeutic administration of SOD@ZIF-8/Mn_3_O_4_ markedly improved the macroscopic appearance of the liver and significantly reduced serum ALT and AST levels, demonstrating its ability to alleviate established APAP-induced hepatic injury (Figure S23, Supporting Information).

These findings, together with the potent antioxidative properties demonstrated earlier, highlight the dual protective role of SOD@ZIF-8/Mn_3_O_4_ in mitigating oxidative stress and inflammation. The hepatoprotective mechanism involves multifaceted modulation of antioxidant defenses, suppression of inflammation, and inhibition of cell death. Moreover, transcriptomic data suggest that the nanozyme reprograms hepatic lipid metabolism related genes, aligning with the observed anti-ferroptotic effect, indicating that its impact extends beyond acute ROS scavenging to long-term restoration of metabolic homeostasis.

### The in vivo safety evaluation of SOD@ZIF-8/Mn_3_O_4_

**Fig. 8:**
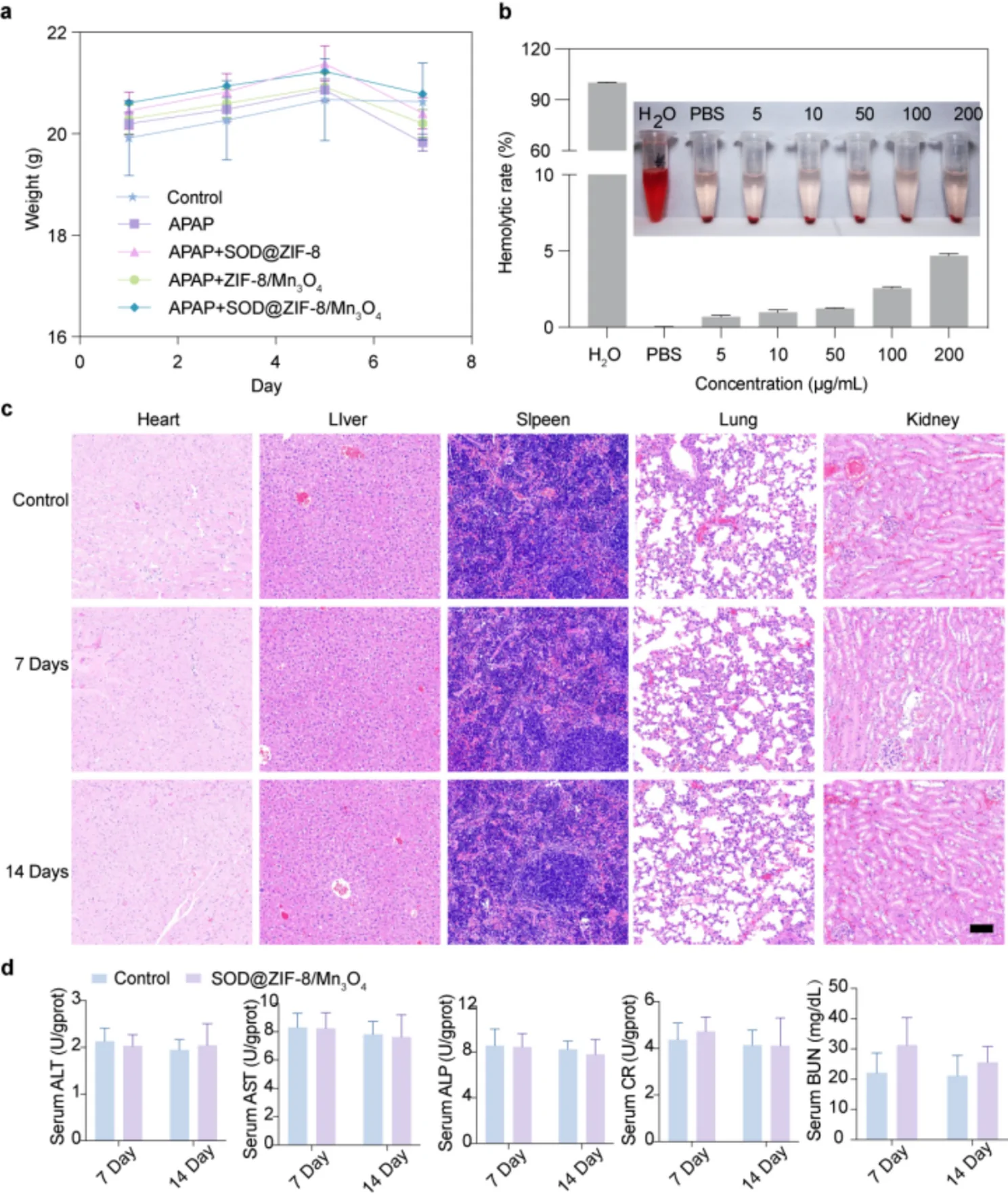
In vivo biosafety of SOD@ZIF-8/Mn_3_O_4_. (**a**) Body weight of the C57 mice in different groups during treatment (*n* = 7). (**b**) In vitro hemolysis assay of SOD@ZIF-8/Mn_3_O_4_ (*n* = 3). (**c**) H&E staining results: in vivo toxicity evaluation of SOD@ZIF-8/Mn_3_O_4_ to major organs (heart, liver, spleen, lung, and kidney) 7 or 14 days after intravenous administration. Scale bars is 100 μm. (**d**) Serum levels of ALT, AST, ALP, CR and BUN of mice were measured 7 or 14 days after intravenous injection of SOD@ZIF-8/Mn_3_O_4_ (*n* = 4). Data presented as mean ± SD

Finally, the in vivo biosafety of SOD@ZIF-8/Mn_3_O_4_ was evaluated in normal mice. As shown in Fig. 8a, the body weights of mice remained stable throughout the administration period, indicating that SOD@ZIF-8/Mn_3_O_4_ did not adversely affect body weight. Hemocompatibility was assessed via a hemolysis assay across a range of nanoparticle concentrations, revealing no observable hemolysis. This result indicates excellent blood compatibility of SOD@ZIF-8/Mn_3_O_4_ (Fig. 8b). Subsequently, in vivo toxicity was examined following intravenous administration of SOD@ZIF-8/Mn_3_O_4_ at a dose of 2 mg/kg. Major organs and blood samples were collected on days 7 and 14 post injection for analysis. H&E staining of major organs revealed no histopathological abnormalities or tissue damage attributable to the nanoparticles (Fig. 8c). Moreover, biochemical analyses of serum ALT, AST, alkaline phosphatase (ALP), creatinine (CR) and blood urea nitrogen (BUN) levels confirmed that hepatic and renal functions remained within normal physiological ranges (Fig. 8d). Taken together, these findings demonstrate the low systemic toxicity and excellent biocompatibility of SOD@ZIF-8/Mn_3_O_4_ in vivo, highlighting its strong potential for therapeutic application in APAP induced liver injury.

## Conclusion

In summary, this study presents the rational design and comprehensive evaluation of a hierarchical nanozyme natural enzyme hybrid platform, SOD@ZIF-8/Mn_3_O_4_, which exhibits robust ROS scavenging, antioxidant enzyme mimicking, and anti lipid peroxidation activities. Unlike conventional single component antioxidants, our system implements a catalytic hierarchy matching strategy that converts the entire pathological ROS cascade (O_2_·^-^converted to H_2_O_2_ converted to ·OH) into benign water, thereby breaking the ROS inflammation feed forward loop. Both in vitro cellular assays and an in vivo mouse model of APAP induced ALI demonstrated the efficacy of this platform in protecting hepatocytes, attenuating inflammation, and improving key liver function biomarkers. Mechanistic investigations through transcriptomic and molecular analyses revealed that the therapeutic benefits are mediated via the modulation of oxidative stress and lipid peroxidation related pathways, including Keap1/Nrf2/HO-1, NF-κB, and GPX4. Importantly, the ZIF-8 framework serves not merely as a carrier but as a spatiotemporal organizer that separates incompatible catalytic steps (SOD inside, Mn_3_O_4_ outside) yet enables substrate channeling. This design principle can be readily extended to other diseases driven by sequential ROS cascades, such as ischemia reperfusion injury, chronic inflammation, and neurodegenerative disorders. This work lays a foundational framework for developing next generation nanomedicines that move beyond passive ROS scavenging toward active pathway reprogramming for oxidative stress related liver pathologies.

## Methods

### Materials

Mn(OAc)_2_·4H_2_O, 2-methylimidazole (2-MIM), Zn(NO_3_)_2_·6H_2_O and APAP were purchased from Sigma-Aldrich Chemical Co. (St. Louis, USA). H_2_O_2_ were provided by Aladdin Industrial Corporation Co., Ltd. (Shanghai, China). 2,7-dichlorofluorescin diacetate (DCFH-DA) and annexin V-FITC/PI Apoptosis Detection Kit for flow cytometry were purchased from Elabscience Biotechnology Co., Ltd. (Wuhan, China). Reverse Transcriptase reagent was purchased from Novoprotein (Suzhou, China).

### Preparation of SOD@ZIF-8/Mn_3_O_4_

First, ZIF-8 nanocarriers were synthesized by dissolving 1.17 g of Zn(NO_3_)_2_·6H_2_O in methanol and 9.85 g of 2-methylimidazole (2-MIM) in methanol separately [35]. The two solutions were then mixed thoroughly and stirred at room temperature for 1 h. The resulting product was washed twice with methanol and dried under vacuum at 50 °C to obtain a powder. To prepare SOD@ZIF-8, a biomimetic mineralization method was employed. Briefly, 5 mg of SOD was dissolved in 2-MIM, and after complete dissolution, Zn(NO_3_)_2_·6H_2_O was added dropwise under gentle stirring at room temperature. The reaction mixture was stirred continuously to allow the formation of SOD@ZIF-8 nanocarriers. The resulting precipitate was collected by centrifugation to remove unencapsulated proteins and finally redispersed in water for further use. Meanwhile, Mn_3_O_4_ nanoparticles were synthesized using a hydrothermal method. Specifically, 0.7 g of Mn(OAc)_2_·4H_2_O was dissolved in DMF [36]. The solution was transferred to a 30 mL Teflon-lined stainless steel autoclave and heated at 130 °C for 8 h. The product was then isolated by repeated centrifugation using ethanol and deionized water, followed by lyophilization to obtain Mn_3_O_4_ nanoparticles in powder form. For the preparation of ZIF-8/Mn_3_O_4_, surface functionalization of Mn_3_O_4_ nanoparticles was first performed by dispersing the synthesized Mn_3_O_4_ (20 mg/mL) in an aqueous 0.5 M citrate solution at pH 7.0 [37]. The suspension was stirred continuously for 12 h, followed by dialysis to remove unbound ligands and impurities. Subsequently, the pre-formed ZIF-8 solution was added at a 1:1 molar ratio, and the mixture was stirred at room temperature for 24 h. The final product, ZIF-8/Mn_3_O_4_, was obtained by sequential washing with ethanol and deionized water, followed by centrifugation. Finally, to obtain SOD@ZIF-8/Mn_3_O_4_, the pre-synthesized SOD@ZIF-8 nanoparticles were mixed with surface-functionalized Mn_3_O_4_, and the mixture was stirred for 24 h at room temperature to allow surface attachment of Mn_3_O_4_ onto SOD@ZIF-8, yielding the final SOD@ZIF-8/Mn_3_O_4_ nanocomposite.

### Preparation of FITC-SOD@ZIF-8/Mn_3_O_4_

Briefly, SOD was first reacted with FITC for 24 h in the dark to obtain FITC-conjugated SOD, with a molar ratio of SOD to probe of 1:3. The resulting solution was dialyzed to remove unreacted dye and impurities. Subsequently, FITC-SOD was used instead of unlabeled SOD and co-mineralized with Zn^2+^ and 2-methylimidazole to prepare FITC-SOD@ZIF-8 through a biomimetic mineralization process. Finally, citrate-modified Mn_3_O_4_ nanoparticles were anchored onto the surface of FITC-SOD@ZIF-8 to obtain FITC-labeled SOD@ZIF-8/Mn_3_O_4_. The obtained samples were collected by centrifugation and washed to remove unbound components before being used in subsequent experiments.

### Characterization of SOD@ZIF-8/Mn_3_O_4_

The morphological evaluation and particle size of the materials were examined using TEM (FEI Tecnai G20 operating at 200 kV, USA), HRTEM (FEI Tal os F200X at 200 kV, USA), and SEM (Zeiss, Gemini 300, Germany). Hydrodynamic size and zeta potential were measured by dynamic light scattering (DLS, Zetasizer Nano ZS, UK). The crystal structure was characterized by X-ray diffraction (XRD, Bruker D8 ADVANCE, Germany), while the molecular structure was analyzed using Fourier transform infrared spectroscopy FT-IR (Thermo Scientific Nicolet IS10, USA). Elemental composition and valence states were investigated by X-ray photoelectron spectroscopy (XPS, Thermo Scientific ESCALAB 250, USA). Surface pore size and specific surface area were determined via nitrogen adsorption–desorption analysis (Micromeritics ASAP 2460, USA). Efficacy in radical scavenging was assessed via EPR (Bruker A300-10, Germany). To assess the long-term stability of SOD@ZIF-8/Mn_3_O_4_ under recommended storage conditions, freshly prepared SOD@ZIF-8/Mn_3_O_4_ was dispersed in DMEM medium supplemented with 10% fetal bovine serum (FBS) at a final concentration of 50 µg/mL. Samples were collected at predetermined time points (days 0, 3, 5, and 7). The hydrodynamic diameter, polydispersity index (PDI), and zeta potential were measured using DLS. All measurements were performed with three replicate runs per sample. Results are presented as mean ± SD.

### Determination of SOD and Mn_3_O_4_ loading content

The Mn^2^⁺ content was determined using inductively coupled plasma optical emission spectrometry (ICP-OES, Thermo ICP-OES 7200, USA). The drug loading and encapsulation efficiency of SOD were calculated by measuring the absorbance at 280 nm using a UV–visible spectrophotometer (UV-vis, SHIMADZU AOE Instruments, Japan), according to the following equations:

$$\begin{aligned}&\:\text{Loading Content (LC}\%) =\\&\frac{\text{Mass of loaded SOD}}{\text{Total mass of nanoparticles}}\times 100\end{aligned}$$

$$\begin{aligned}&\:\text{Encapsulation Efficiency (EE}\%)=\\& \frac{\text{Mass of loaded SOD}}{\text{Initial mass of SOD added}}\times 100\end{aligned}$$

### DFT calculations

Density Functional Theory (DFT) calculations were performed using the VASP software package [38]. The exchange–correlation interactions were treated using the Perdew–Burke–Ernzerhof (PBE) functional within the generalized gradient approximation (GGA). The projector augmented-wave (PAW) method was employed, and the plane-wave kinetic energy cutoff was set to 500 eV. Brillouin zone sampling was carried out using a 5 × 5 × 5 Monkhorst–Pack k-point grid. Geometry optimization was considered complete when all atomic positions were fully relaxed, with the total energy convergence criterion set to 1× 10^− 5^ eV and the force on each atom below 0.03 eV/Å [39, 40].

### Radical scavenging activity assay

The ABTS and DPPH radical scavenging assays were performed following the procedures reported in previous studies [41]. The radical scavenging activity was calculated using the following equation:

$$\:\text{Scavenging Activity}(\%) =\frac{\mathrm{A}\mathrm{(}\mathrm{0}\mathrm{)}\mathrm{-}\mathrm{A}\mathrm{(}\mathrm{sample}\mathrm{)}\text{}}{\mathrm{A}\mathrm{(}\mathrm{0}\mathrm{)}}\times{100}$$

where A(0) represents the initial absorbance of the DPPH or ABTS solution, and A(sample) denotes the absorbance after adding the sample at different concentrations. The hydroxyl radicals (·OH) scavenging activity of SOD@ZIF-8/Mn_3_O_4_ was evaluated according to the manufacturer’s instructions. ·OH were generated through the Fenton reaction between H_2_O_2_ and Fe^2+^. The hydroxyl radical scavenging activity (%) was calculated according to the following formula:

$$\begin{aligned}&\:\cdot\text{OH Scavenging Activity} (\%) = \\&\frac{\mathrm{A}\mathrm{(}\mathrm{sample}\mathrm{)} - \mathrm{A}\mathrm{(control}\mathrm{)}\text{}}{\mathrm{A}\mathrm{(}\mathrm{blank}\mathrm{)}\mathrm{-}\mathrm{A}\mathrm{(control}\mathrm{)}}\times{100}\end{aligned}$$

where Ablank, Asample, and Acontrol represent the absorbance values of the blank group, sample group, and control group, respectively.

The O_2_·^⁻^ scavenging activity of SOD@ZIF-8/Mn_3_O_4_ was also assessed following the manufacturer’s protocol. In this assay, O_2_·^⁻^ were generated by the APS-TEMED system and subsequently reacted with hydroxylamine hydrochloride to produce NO_2_⁻. The superoxide radical scavenging activity (%) was calculated by the following formula:

$$\begin{aligned}&\:\mathrm{O}_2^{\cdot-}\text{Scavengingn Activity}(\%)= \\&\frac{\mathrm{A}\mathrm{(}\mathrm{blank}\mathrm{)}-\mathrm{A}\mathrm{(}\mathrm{sample}\mathrm{)}\text{}}{\mathrm{A}\mathrm{(}\mathrm{blank}\mathrm{)}}\times{100}\end{aligned}$$

### Time-dependent detection of H_2_O_2_-scavenging capacity

To compare the H_2_O_2_-scavenging capacity of SOD@ZIF-8/Mn_3_O_4_ in the presence or absence of GSH, the residual H_2_O_2_ levels in the reaction system were measured at different time points using a hydrogen peroxide assay kit. Briefly, reaction systems containing 200 µM H_2_O_2_ were prepared and divided into the following groups: H_2_O_2_ alone, H_2_O_2_+GSH, H_2_O_2_+SOD@ZIF-8/Mn_3_O_4_, and H_2_O_2_+GSH + SOD@ZIF-8/Mn_3_O_4_. The final concentrations of GSH and SOD@ZIF-8/Mn_3_O_4_ were 1 mM and 50 µg/mL, respectively, and the total reaction volume was 200 µL. The samples were incubated at room temperature and collected at 0, 5, 10, and 30 min. For the groups containing nanomaterials, the samples were centrifuged after incubation to remove the nanoparticles, and the supernatants were used for subsequent analysis. The residual H_2_O_2_ content was determined according to the manufacturer’s instructions of the Beyotime Hydrogen Peroxide Assay Kit by measuring the absorbance at 560 nm. The residual H_2_O_2_ percentage was calculated as follows: residual H_2_O_2_ (%) = C/C0 × 100%, where C0 represents the initial H_2_O_2_ concentration and C represents the H_2_O_2_ concentration detected at each time point. All experiments were performed in triplicate.

### Enzyme like activity of SOD@ZIF-8/Mn_3_O_4_

The SOD-like activity of SOD@ZIF-8/Mn_3_O_4_ was evaluated using the WST-1 method as described by Beijing Solarbio Science&Technology Co., Ltd (China). In this assay, a reaction system containing xanthine and xanthine oxidase generates O_2_·^⁻^, which subsequently reacts with WST-1 to form a water-soluble yellow formazan dye, exhibiting a characteristic absorption peak at 450 nm. The CAT-like activity of SOD@ZIF-8/Mn_3_O_4_ was evaluated using the ammonium molybdate method provided by Nanjing Jiancheng Bioengineering Institute (China). In this assay, H_2_O_2_ reacts with ammonium molybdate to form a stable yellow complex with a maximum absorption peak at 405 nm. The GPX-like activity of SOD@ZIF-8/Mn_3_O_4_ was assessed using the DTNB method, as described by Beijing Solarbio Science & Technology Co., Ltd (China). In this assay, GPX catalyzes the oxidation of reduced glutathione (GSH) by H_2_O_2_ to produce oxidized glutathione (GSSG). GSH reacts with 5,5’-dithiobis-(2-nitrobenzoic acid) (DTNB) to form a yellow-colored compound with a characteristic absorption peak at 412 nm.

### Kinetic analysis of O_2_·^−^ scavenging activity

To characterize the enzyme-like kinetics of SOD@ZIF-8/Mn_3_O_4_ toward its primary substrate O_2_·^−^, kinetic analysis was performed using the WST-1 method. O_2_·^−^ was generated by the xanthine/xanthine oxidase system. Briefly, varying concentrations of xanthine (1, 2, 10, 20, 40, and 80 mM) were prepared in reaction buffer containing WST-1. The reaction was initiated by adding xanthine oxidase in the presence or absence of SOD@ZIF-8/Mn_3_O_4_ (20 µg/mL). The absorbance at 450 nm was monitored continuously for 20 min using a microplate reader. The initial reaction velocity (V_0_) was calculated from the linear portion of the absorbance-time curve (first 5 min) and converted to O_2_·^−^ consumption rate using the molar extinction coefficient of WST-1 formazan (ε = 37,000 M^− 1^·cm^− 1^). The data were fitted to the Michaelis-Menten equation:

$$\:\mathrm{V}\mathrm{=}\frac{\mathrm{Vmax} \times \mathrm{[S]}\text{}}{\mathrm{Km+[S]}}$$

### Cytoprotective effect of SOD@ZIF-8/Mn_3_O_4_ NPs against oxidative stress in vitro

AML-12 cell line obtained from Shanghai ChenYing Biotechnology Co., Ltd (China) was cultured in DMEM/F12 (1:1) (1X) medium, respectively, with 10% FBS, 1% ITS liquid media supplement and 40 ng/mL Dexamethasone in a humidified atmosphere at 37 °C containing 5% CO_2_. The in vitro cytotoxicity of SOD, Mn_3_O_4_, and APAP was assessed using the Cell Counting Kit-8 (CCK-8) assay. The synergistic effects of SOD and Mn_3_O_4_ were evaluated by calculating the combination index (CI) at various concentrations using CompuSyn software and the Chou–Talalay method. To assess lysosomal escape, lysosomes were stained with Lysotracker Red following treatment. Intracellular fluorescence distribution was then visualized using laser confocal microscopy, enabling evaluation of nanoparticle localization and potential escape from lysosomal compartments. ROS levels were assessed using DCFH-DA staining. AML-12 cells were co-incubated with SOD@ZIF-8/Mn_3_O_4_ (10 µg/mL) for 3 h, followed by treatment with APAP for 24 h. After treatment, 10 µM DCFH-DA was added to the cells and incubated for 30 min in the dark. Following incubation, the cells were washed twice with PBS, and intracellular ROS levels were analyzed by fluorescence microscopy and flow cytometry. To specifically evaluate the O_2_·^−^ scavenging capacity of SOD@ZIF-8/Mn_3_O_4_, intracellular O_2_·^−^ levels were measured using MitoSOX Red probes. After treatment, AML-12 cells were incubated with MitoSOX Red. Representative fluorescence images were captured using an inverted fluorescence microscope. All experiments were independently repeated three times, and data are presented as mean ± SD.

### Western blot assay and Real-Time PCR

Total protein was extracted using RIPA buffer supplemented with protease and phosphatase inhibitors. After transfer and blocking, blots were incubated at 4 °C with the following primary antibodies: anti-GPX4 (rabbit, 1:1000, CST), anti-SLC7A11 (rabbit, 1:1000, CST), anti-FSP-1 (rabbit, 1:1000, CST), anti-Nrf2 (rabbit, 1:1000, ABclonal), anti-Keap1 (rabbit, 1:1000, ABclonal), anti-HO-1 (rabbit, 1:1000, ABclonal), anti-p65 (rabbit, 1:1000, CST), anti-p-p65 (rabbit, 1:1000, CST), anti-IκBα (rabbit, 1:1000, CST), anti-p-IκBα (rabbit, 1:1000, CST) and anti-β-Actin (rabbit, 1:1000, CST). Then, protein bands were visualized using the Gel Doc XR + Gel Documentation System (BIO-RAD, USA). After treatment, total RNA was extracted from the cells using TRIzol reagent, following the manufacturer’s protocol. The mRNA expression levels of inflammatory cytokines (IL-6, IL-1β, and TNF-α) were quantified using a real-time fluorescence quantitative PCR (RT-qPCR) kit. Primer sequences used for qPCR are listed in Table S1.

### Validation of SOD@ZIF-8/Mn_3_O_4_-mediated anti-inflammatory mechanism via Nrf2/HO-1 and NF-κB pathway inhibitors

To investigate the necessity of Nrf2 signaling in the protective effects of SOD@ZIF-8/Mn_3_O_4_, intervention experiments were performed using the Nrf2-specific inhibitor ML385 (5 µM). AML-12 cells were divided into five groups: control, APAP model, ML385 alone, APAP + SOD@ZIF-8/Mn_3_O_4_, and APAP + SOD@ZIF-8/Mn_3_O_4_+ML385. Cells were pretreated with ML385 and SOD@ZIF-8/Mn_3_O_4_ for 3 h, followed by incubation with fresh medium containing APAP for an additional 24 h. Cell viability was assessed using the CCK-8 assay, and intracellular ROS levels were measured using the DCFH-DA probe. To determine the involvement of the NF-κB pathway, the NF-κB inhibitor BAY 11–7082 (2 µM) was used. AML-12 cells were divided into five groups: control, APAP model, APAP + BAY 11–7082, APAP + SOD@ZIF-8/Mn_3_O_4_, and APAP + SOD@ZIF-8/Mn_3_O_4_+BAY 11–7082. Treatments were performed as described above, followed by assessment of cell viability and ROS levels. All experiments were independently repeated three times.

### Anti-acute liver injury effects of SOD@ZIF-8/Mn_3_O_4_ NPs in vivo

Male C57BL/6J mice (6–8 weeks old) were purchased from Hangzhou Qizhen Laboratory Animal Co., Ltd. (Zhejiang, China). All animal experiments were conducted in accordance with the guidelines set forth by the Committee for the Protection and Use of Laboratory Animals of Zhejiang Provincial People’s Hospital with ethical approval number 20,241,028,215,720. Mice were housed in groups of five per cage under controlled environmental conditions: ambient temperature of 20–22 °C, relative humidity of 40–60%, and a 12-h light/dark cycle. Animals were provided ad libitum access to standard rodent chow and sterile water. To establish an acute liver injury (ALI) model, mice were fasted for 24 h and subsequently administered a single intraperitoneal injection of APAP at a dose of 300 mg/kg. Hemolysis was assessed by collecting orbital blood samples from mice. The blood was then subjected to centrifugation at 5000 rpm for 10 min. The resulting supernatant was discarded, and various concentrations of SOD@ZIF-8/Mn_3_O_4_ (5, 10, 50, 100, and 200 µg/mL) were added to the erythrocyte suspension. After a 2-h incubation at 37 °C, the absorbances were measured at 450 nm using a microplate reader.

An acute liver injury model was established via intraperitoneal injection of APAP at a dose of 300 mg/kg in a volume of 200 µL, after which the mice were randomly divided into five groups: (1) Normal control group; (2) ALI model group (treated with PBS); (3) 10 mg/kg SOD@ZIF-8 treatment group; (4) 10 mg/kg ZIF-8/Mn_3_O_4_ treatment group; (5) 10 mg/kg SOD@ZIF-8/Mn_3_O_4_ treatment group. Liver tissues were collected after treatment for histological examination to assess tissue damage. Immunofluorescence staining was performed to detect changes in the GPX4 expression. At the conclusion of the treatment period, liver tissues from the ALI model group (PBS treatment) and the SOD@ZIF-8/Mn_3_O_4_ treatment group were collected for transcriptome sequencing.

To compare the therapeutic efficacy of SOD@ZIF-8/Mn_3_O_4_ with the clinically used drug NAC, an APAP-induced acute liver injury model was established in male C57BL/6 mice. Mice were randomly divided into four groups (*n* = 5 per group): (1) Control group, (2) APAP model group, (3) APAP + SOD@ZIF-8/Mn_3_O_4_ group, and (4) APAP + NAC group (150 mg/kg). At 24 h post-APAP administration, liver tissues and serum samples were collected for further analysis. Liver injury was evaluated by macroscopic observation, H&E staining of liver sections, and serum levels of ALT and AST. Then, male C57BL/6J mice were fasted for 24 h and then administered APAP intraperitoneally at 300 mg/kg to induce acute liver injury. Two hours after APAP administration, mice received SOD@ZIF-8/Mn_3_O_4_ intravenously at 10 mg/kg. At 24 h after APAP challenge, blood and liver tissues were collected. Liver injury was evaluated by macroscopic observation, serum levels of ALT and AST. Four mice were included in each group.

### Data processing and statistical analysis

All data are presented as mean±standard deviation (mean ± SD). Statistical analyses were performed using GraphPad Prism version 8.0.2. The normality of data distribution was assessed using the Shapiro-Wilk test. For comparisons between two groups, statistical significance was determined using Student’s t-test. For multiple group comparisons, one-way analysis of variance (ANOVA) was performed. A p-value less than 0.05 (*p* < 0.05) was considered statistically significant, with significance levels denoted as *p < 0.05, **p < 0.01, and ***p < 0.001. All experiments were performed with at least three independent biological replicates, and the exact sample size (n) for each experimental group is clearly indicated in the figure legends.

## Supplementary Information

Supplementary material 2

## Data Availability

No datasets were generated or analysed during the current study.
