# Supplementary material for: Breaking the ROS inflammation loop: a hierarchical nanozyme natural enzyme cascade for acetaminophen induced acute liver injury
Source: J Nanobiotechnology. 2026 Aug 10;24:889. doi: 10.1186/s12951-026-04912-2 (PMC13595542; doi:10.1186/s12951-026-04912-2)
Supplement: Supplementary file 1 [file 12951_2026_4912_MOESM2_ESM.docx]

**Supporting Information**

**Breaking the ROS Inflammation Loop: A Hierarchical Nanozyme Natural Enzyme Cascade for Acetaminophen Induced Acute Liver Injury**

Mengmeng Dong^a,d,1^, Xiaomin Huang^c,1^, Kaiqiang Li^a,d^, Xiongrong Zhu^a,d^, Tingting Hu^a^, Xiaohuan Guo^a^, Ke Hao^a^, Fei Kong^a,d^, Mei Yang^c^, Ning Hu^e^, Yingwei Wang^f^*, Lisen Lu^b^*, Zhen Wang^a,d^*

^a^Laboratory Medicine Center, Department of Transfusion Medicine, Zhejiang Provincial People's Hospital, Affiliated People's Hospital, Hangzhou Medical College, Hangzhou, 310014, China

^b^NHC Key Laboratory of Tropical Disease Control, School of Life Sciences and Medical Technology, Hainan Medical University, Haikou, Hainan, 571199, China

^c^Eye Institute and Department of Ophthalmology, Eye & ENT Hospital, Fudan University, Key Laboratory of Myopia, Chinese Academy of Medical Sciences, Shanghai, 200000, China

^d^School of Laboratory Medicine, Hangzhou Medical College, Hangzhou, 310053, China

^e^General Surgery Department, Children's Hospital, Zhejiang University School of Medicine, National Clinical Research Center for Children's Health, Hangzhou, 310052, China

^f^Center for Laboratory Medicine, Precision Medicine Center，Tiantai People’s Hospital of Zhejiang Province (Tiantai Branch of Zhejiang Provincial People’s Hospital), Hangzhou Medical College, Taizhou, 317200, China

^1^ These authors contributed equally to this work should be considered co-first authors.

^*^Correspondence should be addressed to [yuchen6046@163.com](mailto:yuchen6046@163.com) (Zhen Wang), hy2025061@muhn.edu.cn ( Lisen Lu) and wyw724@163.com (Yingwei Wang)

**Figure S1.** TEM image of SOD@ZIF-8/Mn_3_O_4_.

**Figure S2.** Standard curve for the quantification of SOD loading content. The absorbance at 280 nm was measured to determine the concentration of encapsulated SOD.

**Figure S3.** Colloidal stability of SOD@ZIF-8/Mn_3_O_4_ in different media. The hydrodynamic particle size of SOD@ZIF-8/Mn_3_O_4_ was monitored in H_2_O, PBS, and DMEM at 0, 3, 5, and 7 days.

**Figure S4**. UV-vis absorption spectra of ABTS and DPPH radicals after treatment with ZIF-8/Mn_3_O_4_ and SOD@ZIF-8/Mn_3_O_4_ at varying concentrations. (a, b) ABTS radical-scavenging activity of ZIF-8/Mn_3_O_4_ and SOD@ZIF-8/Mn_3_O_4_. (c, d) DPPH radical-scavenging activity of ZIF-8/Mn_3_O_4_ and SOD@ZIF-8/Mn_3_O_4_.

**Figure S5.** Time-dependent H_2_O_2_-scavenging activity of SOD@ZIF-8/Mn_3_O_4_ in the presence or absence of GSH (n=3). Data are presented as mean ± SD.

**Figure S6.** Time-dependent scavenging of O_2_·^-^, H_2_O_2_, and ·OH by the physically mixed components and SOD@ZIF-8/Mn_3_O_4_. Time-dependent changes in the levels of (a, b) O_2_·^-^, (c, d) H_2_O_2_, and (e, f) ·OH after treatment with the physical mixture or the integrated SOD@ZIF-8/Mn_3_O_4_ nanocomposite (n = 3).

**Figure S7.** Enzyme-mimetic catalytic kinetics of SOD@ZIF-8/Mn_3_O_4_. (a) Michaelis-Menten kinetics and (b) Lineweaver-Burk plot of the SOD-like activity toward O_2_·^-^. (c) Michaelis-Menten kinetics and (d) Lineweaver-Burk plot of the CAT-like activity toward H_2_O_2_. (e) Michaelis-Menten kinetics and (f) Lineweaver-Burk plot of the GPx-like activity with GSH as the variable substrate(n=3). Data are presented as mean ± SD.

**Figure S8.** Z-stack confocal laser scanning microscopy (CLSM) images of FITC-labeled SOD@ZIF-8/Mn_3_O_4_. (A–J) Sequential optical sections taken at different Z-axis positions. (K) Corresponding 3D reconstruction of the Z-stack images, confirming the internalization of the nanocomposite. Scale bar: 50 μm.

**Figure S9.** Cytotoxicity assessment of SOD in AML-12 cells. Cell viability was measured by CCK-8 assay after 24 hours of incubation with varying concentrations of SOD (n = 6). Data are presented as mean ± SD.

**Figure S10.** Cytotoxicity assessment of Mn_3_O_4_ nanoparticles in AML-12 cells. Cell viability was measured by CCK-8 assay after 24 hours of incubation with varying concentrations of Mn_3_O_4_ NPs (n = 6). Data are presented as mean ± SD.

**Figure S11.** Cellular internalization of SOD@ZIF-8/Mn_3_O_4_ in AML-12 cells. CLSM images showing time-dependent uptake of FITC-labeled nanocomposites (green). Nuclei were counterstained with DAPI (blue). Scale bar: 50 μm.

**Figure S12.** Dose-response curve of APAP-induced cytotoxicity in AML-12 cells. Cell viability was assessed by CCK-8 assay after 24 hours of exposure to increasing concentrations of APAP. The half-maximal inhibitory concentration (IC50) was determined to be 1.2 mg/mL. Data are presented as the mean±SD (n = 3).

**Figure S13**. Screening of the working concentration of SOD@ZIF-8/Mn_3_O_4_ in APAP-injured AML-12 cells. SOD@ZIF-8/Mn_3_O_4_ restored cell viability in a concentration-dependent manner, and 10 μg/mL was identified as the lowest concentration producing a significant cytoprotective effect. Data are presented as the mean±SD (n = 3).

**Figure S14.** Combination index (CI) analysis of SOD and Mn_3_O_4_. CI values were calculated using the Chou-Talalay method via CompuSyn software. A CI value of less than 1 at Fa = 0.5 indicates a synergistic interaction between SOD and Mn_3_O_4_.

**Figure S15.** Live/Dead staining of AML-12 cells under different treatment conditions. (a) Representative fluorescence images of cells stained with Calcein-AM (live, green) and propidium iodide (PI, dead, red). Scale bar: 200 μm. (b) Quantitative analysis of live and dead cell fluorescence intensity (n = 3). Data are presented as mean ± SD. Statistical significance was determined by one-way ANOVA (*P < 0.05, **P < 0.01, ***P < 0.001; ns: not significant).

**Figure S16.** Lipid peroxidation detected by BODIPY C11 probe: (a) representative images Scale bar: 200 μm. and (b) quantification (n = 3). Data are presented as mean ± SD. Statistical significance was determined by one-way ANOVA (*P < 0.05, **P < 0.01, ***P < 0.001; ns: not significant).

**Figure S17.** Quantitative analysis of apoptosis in AML-12 cells by flow cytometry (n=3). Data are presented as mean ± SD. Statistical significance was determined by one-way ANOVA (*P < 0.05, **P < 0.01, ***P < 0.001; ns: not significant).

**Figure S18.** Evaluation of cell proliferation by EdU assay in AML-12 cells. (a) Fluorescence micrographs showing EdU-positive cells (proliferating, red) and DAPI-stained nuclei (blue). Scale bar: 200 μm. (b) Quantitative analysis of EdU fluorescence intensity (n = 3). Data are presented as mean ± SD. Statistical significance was determined by one-way ANOVA (*P < 0.05, **P < 0.01, ***P < 0.001).

F**igure S19**. Molecular mechanism of SOD@ZIF-8/Mn_3_O_4_ in inhibiting ferroptosis. (a) Schematic diagram illustrating the proposed mechanism. (b-d) Western blot analysis and quantification of key ferroptosis-related proteins (GPX4, SLC7A11, FSP-1) (n = 3). Data are presented as mean ± SD. Statistical significance was determined by one-way ANOVA (*P < 0.05, **P < 0.01, ***P < 0.001; ns: not significant).

**Figure S20.** Functional validation of Nrf2 and NF-κB pathways in SOD@ZIF-8/Mn_3_O_4_ mediated anti-inflammation using inhibitors. (a–c) Nrf2 inhibition attenuates the protective effects of SOD@ZIF-8/Mn_3_O_4_. (d–f) NF-κB inhibition enhances the antioxidant effect of SOD@ZIF-8/Mn_3_O_4_. a,d. Cell viability was measured by CCK-8 assay. b,e. Fluorescence images of ROS levels detected by DCFH-DA staining. Scale bars: 200 μm. c,f. Relative quantification data (n = 3). Data are presented as mean ± SD. Statistical significance was determined by one-way ANOVA (*P < 0.05, **P < 0.01, ***P < 0.001; ns: not significant).

**Figure S21.** Comparative therapeutic efficacy of SOD@ZIF-8/Mn_3_O_4_ and NAC in APAP-induced ALI mice. (a) Real photo recording and H&E staining of liver. Serum levels of liver function indicators: ALT (b) and AST (c) (n = 5). Data are presented as mean ± SD. scale bar: 50 μm. Statistical significance was determined by one-way ANOVA (*P < 0.05, **P < 0.01, ***P < 0.001; ns: not significant).

**Figure S22**. Immunofluorescence staining of GPX4 in liver tissues from different treatment groups (scale bar: 50 μm).

**Figure S23.** Therapeutic efficacy of SOD@ZIF-8/Mn_3_O_4_ in mice with APAP-induced acute liver injury. (a) Experimental timeline of APAP challenge followed by therapeutic administration of SOD@ZIF-8/Mn_3_O_4_. (b) Representative macroscopic appearance of liver tissues from the different treatment groups. Serum levels of (c) ALT and (d) AST in the different groups. Data are presented as the mean±SD (n =4).

Table S1 Primer sequences used for quantitative real-time PCR (qPCR).

|  | Forward primer sequences | Reverse primer sequences |
| --- | --- | --- |
| GAPDH | TCAACAGCAACTCCCACTCTTCCA | ACCTGTTGCTGTAGCCGTATTCA |
| IL-1β | GAAATGCCACCTTTTGACAGTG | TGGATGCTCTCATCAGGACAG |
| IL-6 | CTGCAAGAGACTTCCATCCAG | AGTGGTATAGACAGGTCTGTTGG |
| TNF-α | CAGGCGGTGCCTATGTCTC | CGATCACCCCGAAGTTCAGTAG |

Table S2 Surface area and pore volume of ZIF-8 and ZIF-8/Mn_3_O_4_ measured by nitrogen adsorption-desorption analysis

|  | Surface areas (m^2^g^-1^) | Pore volume (cm^3^g^-1^) |
| --- | --- | --- |
| ZIF-8 | 1,386.41 | 1.036489 |
| ZIF-8/Mn_3_O_4_ | 877.5316 | 0.68477 |

Table S3 Storage stability of SOD@ZIF-8/Mn_3_O_4_

| Time (day) | Size (nm) | PDI | Zeta potential (mV) |
| --- | --- | --- | --- |
| 0 | 143.13 ± 3.81 | 0.18 ± 0.07 | -28.11 ± 0.78 |
| 3 | 135.64 ± 1.30 | 0.21 ± 0.02 | -29.02 ± 0.29 |
| 5 | 149.94 ± 2.93 | 0.28 ± 0.01 | -29.65 ± 0.90 |
| 7 | 155.40 ± 1.40 | 0.26 ± 0.04 | -30.85 ± 0.53 |
